# VaRank: a simple and powerful tool for ranking genetic variants

**DOI:** 10.7717/peerj.796

**Published:** 2015-03-03

**Authors:** Véronique Geoffroy, Cécile Pizot, Claire Redin, Amélie Piton, Nasim Vasli, Corinne Stoetzel, André Blavier, Jocelyn Laporte, Jean Muller

**Affiliations:** 1 Laboratoire de Génétique médicale, UMR_S INSERM U1112, IGMA, Faculté de Médecine FMTS, Université de Strasbourg, Strasbourg, France; 2 IGBMC, CNRS UMR 7104/INSERM U964/Université de Strasbourg, Illkirch Cedex, France; 3 Laboratoire de Diagnostic Génétique, Hôpitaux Universitaires de Strasbourg, Strasbourg Cedex, France; 4 Interactive Biosoftware, Rouen, France; 5 Laboratoire ICUBE, UMR CNRS 7357, LBGI, Université de Strasbourg, Strasbourg, France

**Keywords:** Next generation sequencing, Variant ranking, Human genetics, Molecular diagnostic, Mutation detection, Annotation, Software, Barcode

## Abstract

**Background.** Most genetic disorders are caused by single nucleotide variations (SNVs) or small insertion/deletions (indels). High throughput sequencing has broadened the catalogue of human variation, including common polymorphisms, rare variations or disease causing mutations. However, identifying one variation among hundreds or thousands of others is still a complex task for biologists, geneticists and clinicians.

**Results.** We have developed VaRank, a command-line tool for the ranking of genetic variants detected by high-throughput sequencing. VaRank scores and prioritizes variants annotated either by Alamut Batch or SnpEff. A barcode allows users to quickly view the presence/absence of variants (with homozygote/heterozygote status) in analyzed samples. VaRank supports the commonly used VCF input format for variants analysis thus allowing it to be easily integrated into NGS bioinformatics analysis pipelines. VaRank has been successfully applied to disease-gene identification as well as to molecular diagnostics setup for several hundred patients.

**Conclusions.** VaRank is implemented in Tcl/Tk, a scripting language which is platform-independent but has been tested only on Unix environment. The source code is available under the GNU GPL, and together with sample data and detailed documentation can be downloaded from http://www.lbgi.fr/VaRank/.

## Introduction

In recent years, high throughput sequencing has generated thousands of new genomes from various species across the tree of life and millions of genetic variants. Especially in the field of human genetics, targeted or whole exome and genome sequencing are becoming standard assays (Ng et al., 2010; Ng et al., 2009; Saunders et al., 2012) to identify causal single-nucleotide variations (SNVs) as well as short insertions/deletions (indels) in patients with Mendelian diseases, or variants associated to increased disease risk (Cirulli & Goldstein, 2010; Manolio et al., 2009).

**Figure 1:**
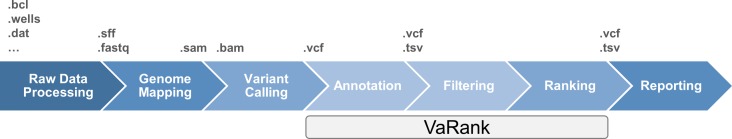
High throughput sequencing data analysis workflow and VaRank positioning.

The classical data workflow in next generation sequencing includes several bioinformatics steps from the raw sequencing data analysis which transforms the signal from the sequencers (e.g., fluorescence, pH…) to raw sequences that are further aligned to the reference genome. Sequence differences from the reference genome (variants) are then detected aiming at identifying causal mutation (Fig. 1). Although sequencing limitations are overcome with increasing instrument capacity (Glenn, 2011 and http://www.molecularecologist.com/next-gen-fieldguide-2014/), the development of bioinformatics solutions for variant prioritization remains a great challenge. The focus on high throughput sequencing resulted in the development of a variety of tools, protocols and applications including variant filtering and ranking (for a review see Bao et al., 2014). Recent approaches include the use of additional data such as haploinsufficiency prediction and phenotype information (Sifrim et al., 2013), cross species phenotype information (Robinson et al., 2014) or interaction data (Smedley et al., 2014) to enhance the analysis. However, molecular biologists still require simple tools in the variant filtering and ranking process to identify causal mutations among a large pool of rare variants existing in each individual.

**Figure 2:**
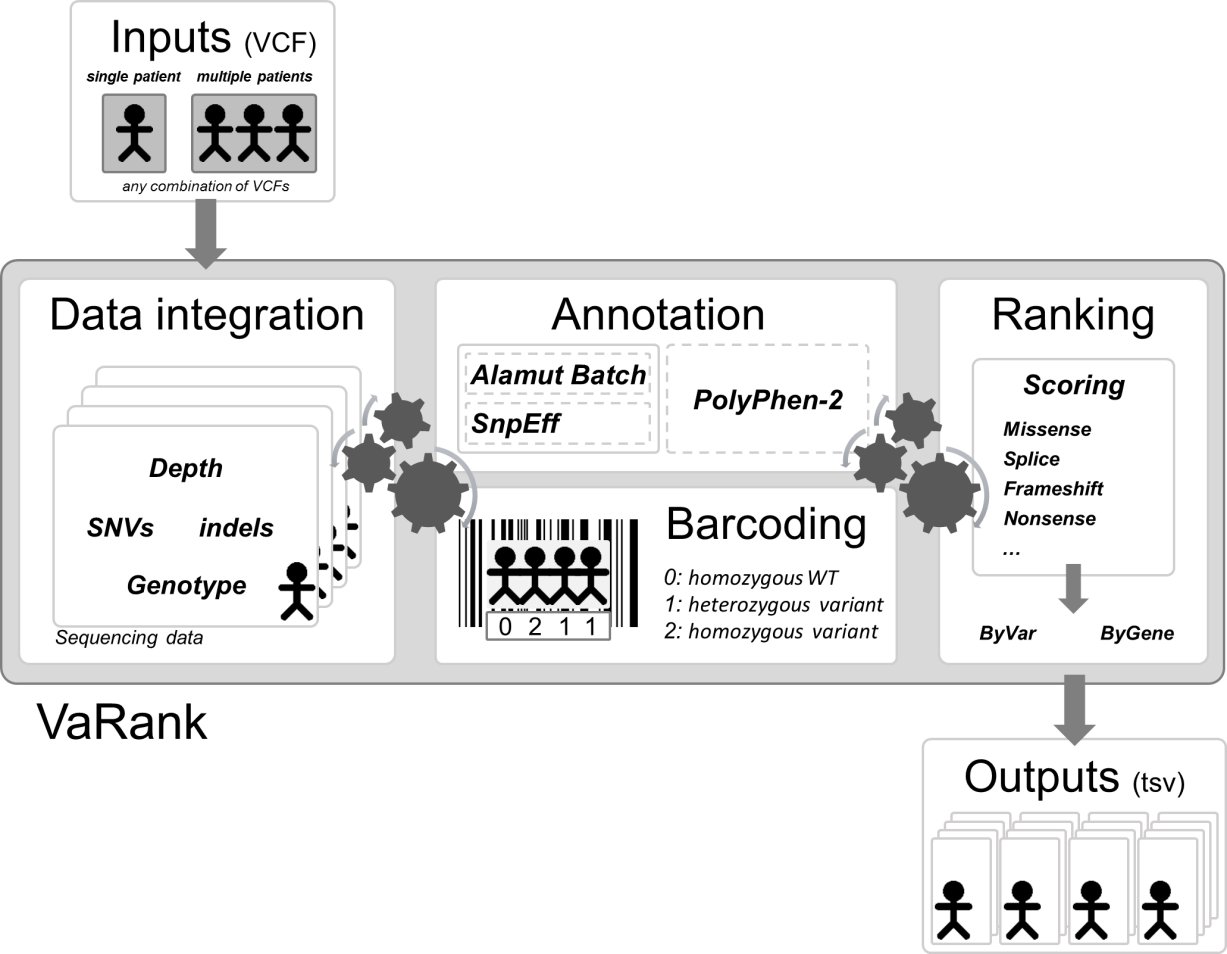
VaRank’s workflow. The work flow is separated into 4 major steps, (i) Sequencing data from a single or from multiple VCF files are integrated including variant call quality summary, (ii) Annotation of each variant including genetic and predictive information (functional impact, putative effects in protein coding regions, population frequency, phenotypic features…) from different sources. The annotation can either be done by Alamut Batch or SnpEff. (iii) Presence/absence of variants (with homozygote/heterozygote status) within all samples represented in a barcode, and (iv) Prioritization, to score and rank variants according to their predicted pathogenic status. The final output files are available for each samples.

Here we propose a new simple and powerful tool named VaRank (http://www.lbgi.fr/VaRank) for human variant ranking which provides a comprehensive workflow for annotating and ranking SNVs and indels. Four modules create the strength of this workflow (Fig. 2): (i) Data integration with variant call quality summary, to filter out false positive calls, depending on the sequencing technology and the analysis pipeline; (ii) Variant annotation to integrate genetic and predictive information (functional impact, putative effects in protein coding regions, population frequency, phenotypic features…) from different sources, using HGVS nomenclature (Taschner & den Dunnen, 2011); (iii) Presence/absence of variants (with homozygote/heterozygote status) within all samples represented in a barcode, to search for recurrence between families or group of individuals and (iv) Prioritization, to score and rank variants according to their predicted pathogenic status.

VaRank can substantially reduce the number of potential causal variants to be manually inspected for further studies and increase the efficiency of sequence analysis for researchers and clinicians. VaRank has been already successfully applied in the field of human genetics, first in research including identification of new genes responsible for rare human disease and second in diagnostics to identify mutation in known human disease implicated genes. In this work we describe VaRank as a tool and how it is implemented but also present how it was used in several real datasets.

## Implementation

VaRank is written in Tcl/Tk and runs on all Unix platforms with a standard Tcl/Tk 8.5 installation and one of the compatible annotation engines (Alamut Batch, a commercial software developed by Interactive Biosoftware, Rouen, France, or SnpEff (Cingolani et al., 2012)). PolyPhen-2 (Adzhubei et al., 2010) can also be installed locally and results can be automatically integrated into VaRank.

### Input and output

To run VaRank, the user specifies the input files and chosen options with a single command. In case of wrong commands, VaRank will print a description of the options and defaults settings.

VaRank reads SNV and indel variant descriptions from a VCF file (variant call format Danecek et al., 2011), the reference format for genomic variants. The VCF file can be gzip compressed. The program can take any combination of a single VCF file with multiple patients and/or multiple VCF files with single patient’s data as input (Fig. 2).

Each variant is checked for consistency (genotype, depth of coverage, variant call especially for indels) and VaRank prints warnings if appropriate (i.e., patients redundancy based on the sample identifiers in the VCFs), information from each analysis step (including status and running time), as well as statistics on the submitted data such as total number of variants and number of patients.

The output from VaRank is presented in an easily-accessible tab-separated file that can be opened in any spreadsheet program. Output files report one variant per line. Nevertheless, two types of rankings are provided: one presenting each variant independently, ordered from most likely pathogenic to least likely pathogenic (files with “byVar” suffix), and another ranking genes from most likely causative to least likely causative (files with “byGene” suffix). For the latter, each gene is scored along two criteria: (i) based on its homozygous most pathogenic variant, or (ii) based on its first two heterozygous most pathogenic variants. In order to make sure that no variants are overlooked by the user (by only displaying the most pathogenic variants) all other gene variants are also reported. The “byVar” file is more appropriate for analyzing patient’s data under the dominant or pseudominant hypothesis, while the “byGene” file is more appropriate for recessive diseases (especially for compound heterozygous cases).

Each of these output files is also available in two versions: one contains all submitted variants (“AllVariants” files) while the other one is prefiltered (“filteredVariants” files). The default filters remove variants: (i) called with a read depth <=10, (ii) with a supporting read count <=10, (iii) with a ratio of supporting reads <=15%, (iv) with a validated status annotation in the dbSNP (Sherry et al., 2001) database (based on at least two supporting evidences) that are not pathogenic (based on the ClinicalSignificance field), and (v) with an allele frequency >1% (extracted from the dbSNP database or the Exome Variant Server). All these parameters can be modified by the user.

Finally, a short report of counts (homozygous, heterozygous and total counts) for each of the variant categories (5′ and 3′ UTR, upstream, downstream, frameshift, in-frame, nonsense, splice site, start loss, stop loss, missense, synonymous, intronic, not annotated) is generated for each sample and for the whole submitted dataset.

### Variant annotation

**Figure 3:**
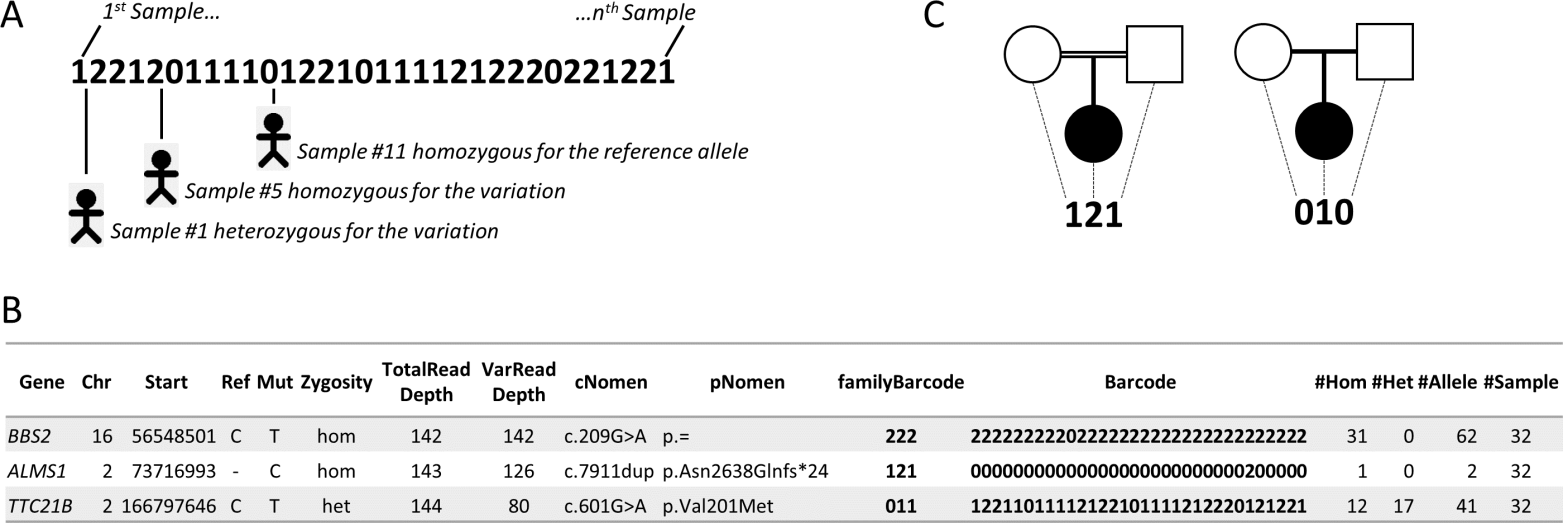
Barcode. (A) The barcode represents the SNV’s zygosity status in an ordered list of samples. Samples that are homozygous for the reference allele are represented using “0,” heterozygous variants are represented using “1” and homozygous variants are represented with “2.” (B) Selected annotations from the VaRank output representing 3 variants from a single patient. The barcode gives an overview of the presence/absence of one variant in all other patients analyzed. The family barcode gives a user ordered view of the presence/absence of one variant in a selection of patients. Together with this, the total counts of alleles are given in the last 4 columns. (C) Example of pedigrees and barcodes that can be specifically used in family analyses such as trio exome sequencing. On the left, homozygous mutations in a consanguineous family could be highlighted by the “121” barcode indicating homozygous variants (“2”) in the proband inherited from heterozygous parents (“1”). On the right de novo variants in the proband could be highlighted with the proposed barcode “010.”

The annotation of variants is performed by the annotation engine (Fig. 2). It is composed of several parts including the main annotation software, which can be either Alamut Batch or SnpEff, the barcode annotation (see Fig. 3 and corresponding section) and optionally PolyPhen-2 predictions. As an example, Alamut Batch collects among others (Table 2): the gene symbol, the OMIM ID, the transcript ID (i.e., RefSeq), the protein IDs (RefSeq and UniProt), the HGVS nomenclature (genomic, cDNA and proteic), information from public variation databases such as dbSNP and the Exome Variant Server (EVS, http://evs.gs.washington.edu/EVS/) and predicted effects at both nucleotide and protein levels. When available, known mutations are highlighted by extracting either reported SNVs/indels flagged as “probably-pathogenic”/“pathogenic” in the field “Clinical significance” introduced since dbSNP134 or using the HGMD database (Stenson et al., 2014).

The choice of transcript is a critical task that can lead to misannotation (McCarthy et al., 2014). To avoid underestimating variant effects, they are annotated on all transcripts available (i.e., one variant can be either intronic or exonic depending on the isoform) and the most pathogenic effect is retained. Given that VaRank is compatible with 2 annotation software there are small differences. Using Alamut Batch, each variant is scored for each transcript. By default we report the longest transcript for each gene except if any variation is more pathogenic in another transcript. In the case of SnpEff, the annotations are already sorted from the most to the least pathogenic.

In order to further enrich the annotation for each variant and each gene, VaRank can integrate (using the option -extann) external annotations provided by the user as a tab separated file. One could for instance associate private expression data or transmission mode for each gene of interest.

**Figure 4:**
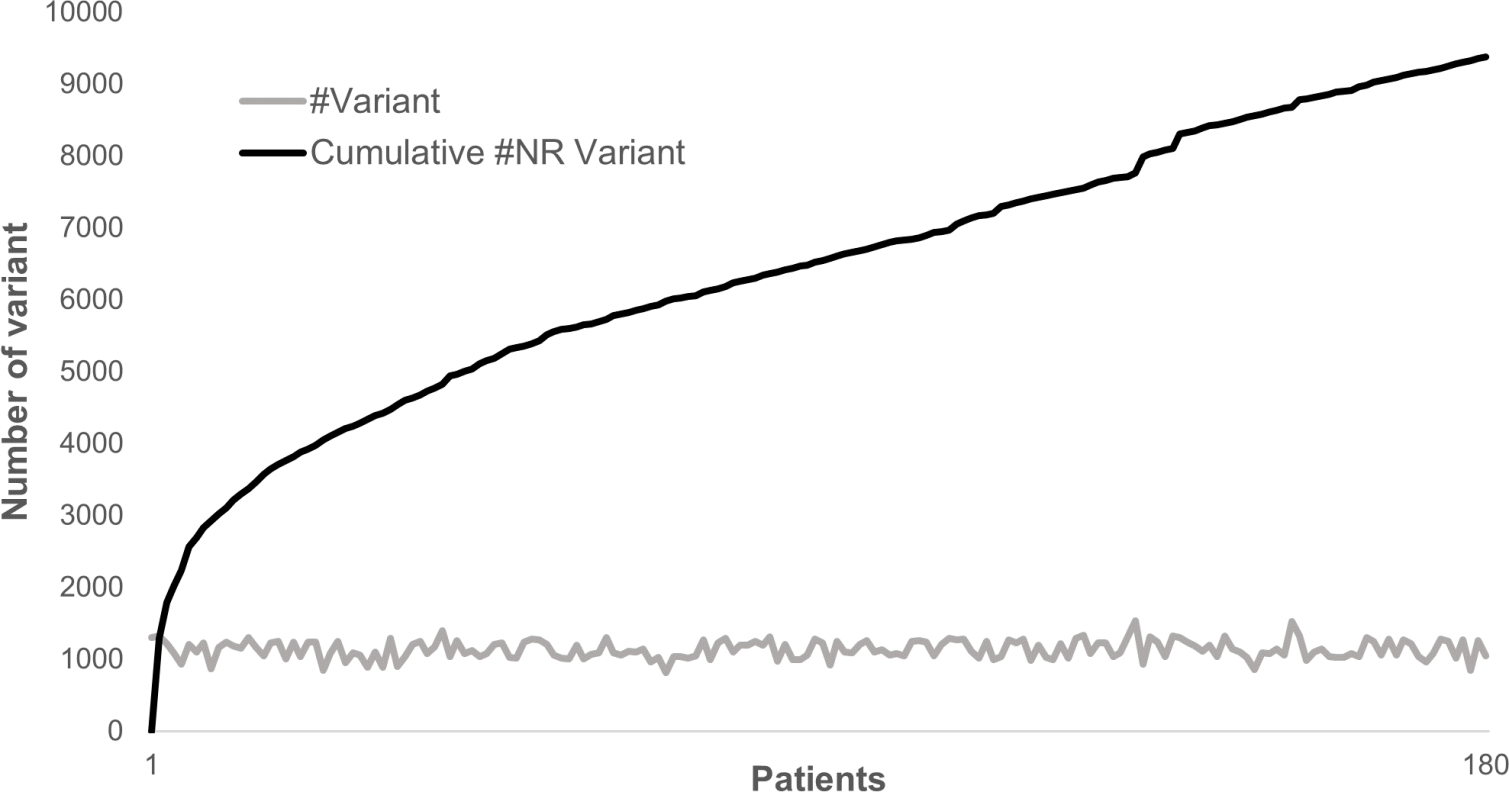
Distribution of variants in 180 patients for 217 genes. The gray line represents the distribution of the number of variants identified in each sample in a cohort of 180 patients sequenced for 217 genes. The dark line represents the cumulative number of non-redundant (NR) variants in the same dataset due to each new sample added.

Annotating and analyzing several individuals together can be very computationally effective since most of the variants are common polymorphisms. As an example, looking at 180 patients sequenced for 217 genes, a total of 204,625 variations could be identified where only 9,378 were non redundant. In this case, the separate annotation of each patient’s variant set would have required ∼20× times the computational cost of the combined analysis. Although the total number of non-redundant variants does not plateau, each new sample adds only a very limited number of new variants to the analysis (Fig. 4).

### Variant ranking

The observed variants (SNVs/indels) can be characterized at different levels (DNA, RNA and protein levels) that VaRank aims at summarizing into a single score. This score is then used to rank variants based on their predicted pathogenicity and thus accelerates identification of relevant ones by biologists. The aim of this score is not to provide yet another score to assess the pathogenicity of each variant but a rationale to present the most relevant variants according to the biologist common use and interpretation rules. Thus, the relative weights of each score components were determined experimentally to best separate categories. VaRank uses the variation type (i.e., substitution, deletion, insertion, duplication) and the coding effect to score. The VaRank scoring is categorized from the most likely to the less likely pathogenic state as follows (score in parenthesis): known mutation (110), nonsense (100), frameshift (100), start loss (80), stop loss (80), missense (50), in-frame (40) and synonymous coding (10) (Table 1).

**Table 1:** Scoring scheme description. Scores in bold reflect score values after the adjustment score is applied.

Variant category	Option name	VaRank score	Definitions
Known mutation	*S_Known*	110	Known mutation as annotated by HGMD and/or dbSNP (rsClinicalSignificance = “pathogenic/probable-pathogenic”).
Nonsense	*S_Nonsense* ^a^	100, **105**	A single-base substitution in DNA resulting in a STOP codon(TGA, TAA or TAG).
Frameshift	*S_Fs*	100	Exonic insertion/deletion of a non-multiple of 3bp resulting often in a premature stop in the reading frame of the gene.
Essential splice site	*S_EssentialSplice* ^a^	90, **95**	Variation in one of the canonical splice sites resulting in a significant effect on splicing.
Start loss	*S_StartLoss* ^a^	80, **85**	Variation leading to the loss of the initiation codon (Met).
Stop loss	*S_StopLoss* ^a^	80, **85**	Variation leading to the loss of the STOP codon.
Intron-exon boundary	*S_CloseSplice* ^a^	70, **75**	Variation outside of the canonical splice sites (donor site is −3 to +6, acceptor site −12 to +2).
Missense	*S_Missense* ^a^ ^,^ ^b^	50, **55, 60, 65**	A single-base substitution in DNA not resulting in a change in the amino acid.
Indel in-frame	*S_Inframe*	40	Exonic insertion/deletion of a multiple of 3bp.
Deep intron-exon boundary	*S_DeepSplice* ^a^	25, **30**	Intronic variation resulting in a significant effect on splicing.
Synonymous coding	*S_Synonymous* ^a^	10, **15**	A single-base substitution in DNA not resulting in a change in the amino acid.

**Notes.**

a Each variant score is adjusted (+5) if high conservation at the genomic level is observed (phastCons cutoff >0.95).

b Missense scores are adjusted (+5) for each deleterious prediction (SIFT and/or PPH2).

**Table 2:** Summary description of the annotations provided by VaRank using Alamut Batch.

Column name	Annotation
VariantID	Variant identifier [#chr]_[genomicposition]_[RefBase]_[VarBase]
Gene	Gene symbol
omimId	OMIM^®^ id
TranscriptID	RefSeq transcript id
TranscriptLength	Length of transcript (full cDNA length)
Chr	Chromosome of variant
Start	Start position of variant
End	End position of variant
Ref	Nucleotide sequence in the reference genome (restricted to 50bp)
Mut	Alternate nucleotide sequence (restricted to 50bp)
Uniprot	Uniprot
protein	Protein id (NCBI)
posAA	Amino acid position
wtAA_1	Reference codon
varAA_1	Alternate codon
Phred_QUAL	QUAL: The Phred scaled probability that a REF/ALT polymorphism exists at this site given sequencing data. Because the Phred scale is −10 * log(1 − *p*), a value of 10 indicates a 1 in 10 chance of error, while a 100 indicates a 1 in $10\hat {\hspace{0.167em} }10$ chance. These values can grow very large when a large amount of NGS data is used for variant calling.
HomHet	Homozygote or heterozygote status
TotalReadDepth	Total number of reads covering the position
VarReadDepth	Number of reads supporting the variant
%Reads_variation	Percent of reads supporting variant over those supporting reference sequence/base
VarType	Variant Type (substitution, deletion, insertion, duplication, delins)
CodingEffect	Variant Coding effect (synonymous, missense, nonsense, in-frame, frameshift, start loss, stop loss)
VarLocation	Variant location (upstream, 5’UTR, exon, intron, 3’UTR, downstream)
Exon	Exon (nearest exon if intronic variant)
Intron	Intron
gNomen	Genomic-level nomenclature
cNomen	cDNA-level nomenclature
pNomen	Protein-level nomenclature
rsID	dbSNP variation
rsValidation	dbSNP validated status
rsClinicalSignificance	dbSNP variation clinical significance
rsAncestralAllele	dbSNP ancestral allele
rsHeterozygosity	dbSNP variation average heterozygosity
rsMAF	dbSNP variation global Minor Allele
rsMAFAllele	dbSNP variation global minor allele
rsMAFCount	dbSNP variation sample size
1000g_AF	1,000 genomes global allele frequency
1000g_AFR_AF	1,000 genomes allele frequency in African population
1000g_SAS_AF	1,000 genomes allele frequency in South Asian population
1000g_EAS_AF	1,000 genomes allele frequency in East Asian population
1000g_EUR_AF	1,000 genomes allele frequency in European population
espRefEACount	ESP reference allele count in European American population
espRefAACount	ESP reference allele count in African American population
espRefAllCount	ESP reference allele count in all population
espAltEACount	ESP alternate allele count in European American population
espAltAACount	ESP alternate allele count in African American population
espAltAllCount	ESP alternate allele count in all population
espEAMAF	Minor allele frequency in European American population
espAAMAF	Minor allele frequency in African American population
espAllMAF	Minor allele frequency in all population
espAvgReadDepth	Average sample read Depth
delta MESscore (%)	% difference between the splice score of variant with the score of the reference base
wtMEScore	WT seq. MaxEntScan score
varMEScore	Variant seq. MaxEntScan score
delta SSFscore (%)	% difference between the splice score of variant with the score of the reference base
wtSSFScore	WT seq. SpliceSiteFinder score
varSSFScore	Variant seq. SpliceSiteFinder score
delta NNSscore (%)	% difference between the splice score of variant with the score of the reference base
wtNNSScore	WT seq. NNSPLICE score
varNNSScore	Variant seq. NNSPLICE score
DistNearestSS	Distance to Nearest splice site
NearestSS	Nearest splice site
localSpliceEffect	Splicing effect in variation vicinity (New donor Site, New Acceptor Site, Cryptic Donor Strongly Activated, Cryptic Donor Weakly Activated, Cryptic Acceptor Strongly Activated, Cryptic Acceptor Weakly Activated)
SiftPred	SIFT prediction
SiftWeight	SIFT score ranges from 0 to 1. The amino acid substitution is predicted damaging is the score is <=0.05, and tolerated if the score is >0.05.
SiftMedian	SIFT median ranges from 0 to 4.32. This is used to measure the diversity of the sequences used for prediction. A warning will occur if this is greater than 3.25 because this indicates that the prediction was based on closely related sequences. The number should be between 2.75 and 3.5
PPH2pred	PolyPhen-2 prediction using HumVar model are either “neutral, possibly damaging, probably damaging” or “neutral, deleterious” depending on the annotation engine.
phyloP	phyloP
PhastCons	PhastCons score
GranthamDist	Grantham distance
VaRank_VarScore	Prioritization score according to VaRank
AnnotationAnalysis	Yes or No indicates if the variation could annotated by any annotation engine
Avg_TotalDepth	Total read depth average at the variant position for all samples analyzed that have the variation
SD_TotalDepth	Standard deviation associated with Avg_TotalDepth
Count_TotalDepth	Number of samples considered for the average total read depth
Avg_SNVDepth	Variation read depth average at the variant position for all samples analyzed that have the variation
SD_SNVDepth	Standard deviation associated with Avg_SNVDepth
Count_SNVDepth	Number of samples considered for the average SNV read depth
familyBarcode	Homozygote or heterozygote status for the sample of interest and its associated samples
Barcode	Homozygote or heterozygote status for all sample analyzed together (Hom: 2; Het: 1; Sample name is given at the first line of the file: ## Barcode)
Hom_Count	Number of homozygote over all samples analyzed together
Het_Count	Number of heterozygote over all samples analyzed together
Allele_Count	Number of alleles supporting the variant
Sample_Count	Total number of samples

In addition when using Alamut Batch, each variant is assessed for any potential effect on the nearest splice site. Following the guidelines from Houdayer et al. (2012) and our own tests (data not shown), we selected three assessment programs: MaxEntScan (Yeo & Burge, 2004), NNSplice (Reese et al., 1997) and Splice Site Finder (based on Shapiro & Senapathy, 1987). A variant is considered to affect splicing when at least two out of the three programs indicate a significant score change between the wild type and the mutated sequences (respectively −10%, −5% and −15%). A VaRank score is then attributed depending on the following three splice site categories (score into parenthesis): (i) essential splice site: two first intronic bases up- or downstream the exon (90), (ii) intron-exon boundary: donor site from −3 to +6, acceptor site from −12 to +2 (70) and (iii) deep intronic changes (25). It should be noted that a SNV affecting the first or last base of an exon can either have a coding effect as a missense or an effect on splicing. VaRank reports the most pathogenic of these two possible effects.

If appropriate the variant score is further adjusted using additional information as nucleotide-level conservation (phastCons Siepel et al., 2005) and protein-level pathogenicity predictions (SIFT and PolyPhen-2) (Adzhubei et al., 2010; Kumar, Henikoff & Ng, 2009) that are used to compute an adjustment score (0 or +5) to be added to the relevant category (Table 1).

### Barcode

Comparing variations of several individuals, related and/or unrelated, affected or not, has proven to be a very effective strategy for distinguishing polymorphisms from variants causing or increasing the likelihood of disease (Ng et al., 2010; Ng et al., 2009). In order to take advantage of this, VaRank introduces a barcode that allows a quick overview of the presence/absence status of each variant within all samples and their zygosity status (“0” representing homozygous wild type, “1” heterozygous and “2” homozygous for the variant) (Fig. 3A). In Fig. 3B, three variants are reported for one specific patient out of a cohort of 32 samples analyzed together. For example, the third variation c.601G>A in *TTC21B* is heterozygous for this patient. In light of the presented barcode, one can immediately notice that the variant is also present in 28 other samples from the cohort, of which in total 12 are homozygous and 17 heterozygous.

In order to allow inheritance analysis, a second barcode (the family barcode) representing only user selected samples can be defined. As an example in trio exome sequencing, we have represented two typical pedigrees (Figs. 3B and 3C), one consanguineous family on the left and one sporadic case on the right. In the case of consanguinity, homozygous mutations are often the cause of the disease in the family. This could be highlighted by selecting the “121” barcode indicating homozygous variants (“2”) in the proband inherited from heterozygous parents (“1”). In the sporadic case, several hypotheses could be tested including a *de novo* variant which could be highlighted using the barcode “010.”

Together with the barcode, simple counts on the individuals (homozygous, heterozygous and total allelic counts) are also available and can easily be used to further filter variants. Indeed, in rare diseases such as the Bardet-Biedl syndrome (BBS, OMIM# 209900), mutations are often private (i.e., one mutation found only in one family) (Muller et al., 2010) meaning that their frequency in the population is very low. Counts can be used to estimate the frequency of a known variant in the user cohort and add significant value to variants not yet reported in any public variant database but for which a frequency can be estimated based on the user’s cohort. As an example, looking at 2,888 non redundant SNVs observed in 107 patients with moderate to severe intellectual disability, 979 did not have any frequency information in the dbSNP and EVS databases. Such information could be directly retrieved from the VaRank output.

The observed frequency of variants in public databases but also in private cohorts can be a powerful filtering strategy. Using the same data (Fig. 3B), variant c.7911dup in *ALMS1* is present only once in the cohort of patients at the homozygous state and is very likely the disease causing mutation in this patient.

## Results and Discussion

VaRank was successfully applied in various human genetics studies both in diagnostics and research. In total, more than 800 patients from several datasets of increasing complexity including the Cockayne syndrome (10 genes tested, manuscript in preparation), Bardet-Biedl syndrome (30 genes (Redin et al., 2012)), ataxias (60 genes), leucodystrophies (70 genes, manuscript in preparation), congenital myopathies (142 genes (Vasli et al., 2012) and 275 genes, manuscript in preparation), intellectual disability (217 genes, Redin et al., 2014) and exome sequencing (Scheidecker et al., 2014) have been analyzed to highlight potential pathogenic variants.

In the following sections, we will provide insight into several datasets analyzed by VaRank and that were used to validate the tool and to highlight its effectiveness. All the input files and output files from the following datasets are available online (www.lbgi.fr/VaRank).

### Bardet-Biedl syndrome (BBS) dataset

The Bardet-Biedl syndrome (BBS; OMIM# 209900) is a pleiotropic recessive disorder, part of the ciliopathies, characterized by extensive genetic heterogeneity counting to date 19 genes (Aldahmesh et al., 2014; Scheidecker et al., 2014). We applied targeted high-throughput sequencing for 30 ciliopathy related genes to 52 patients with clinical features compatible with BBS (Redin et al., 2012). VaRank was used to annotate and rank the variants identified in those patients. Thirty-two cases could be solved by this approach leading to frameshift, missense and splice site mutations all validated by Sanger sequencing (we excluded Copy Number Variations). Sequencing data from the 32 positive samples have been reanalyzed using Alamut Batch version 1.1.11 and PolyPhen-2 v2.2.2 installed on our local servers. A total of 784 non redundant variants have been annotated resulting on average into 167 private variants per sample. We extracted the validated mutations and highlighted the ranking position in the output files (Table 3). In 30/32 samples, mutations were ranked first, while in the remaining ones they were present in the top five among ∼170 variants per patient. This result clearly shows the effectiveness of the ranking in such situation.

**Table 3:** Analysis of 32 patients with Bardet-Biedl syndrome. From the BBS dataset, mutations and ranking for 32 patients sequenced for 30 genes. The ranking for each mutation has been obtained from the “filteredVariants” output. Mutations in italics are predicted to affect splicing.

Patient#	Gene	RefSeq	Mutation (cDNA)	Mutation (protein)	Ranking
P11	***BBS1***	NM_024649.4	c.[436C>T];[436C>T]	p.[R146*];[R146*]	Rank 1
ALD6^a^			c.[436C>T];[(592-?)_(830+?)del]	p.[R146*];[ ?]	Rank 1
ALO47			*c.[479G>A];[479G>A]*	p.[R160Q];[R160Q]	Rank 1
AIO57			c.[670G>A];[670G>A]	p.[E224K];[E224K]	Rank 1
AMK19			c.[951+1G>A];[1169T>G]	p.[?];[M390R]	Rank 1, Rank 2
P9			*c.[1110G>A];[1110G>A]*	p.[ ?];[ ?]	Rank 1
AKH61			c.[1169T>G];[1169T>G]	p.[M390R];[M390R]	Rank 1
P1			*c.[1471*+*4G*>*A]*; *[1471*+*4G*>*A]*	p.[?];[?]	Rank 2^c^
AHZ63^b^			c.[1473+4T>A];[=]	p.[?];[=]	Rank 1
AGA99	***BBS2***	NM_031885.3	*c.[118-1G*>*C];[118-1G*>*C]*	p.[ ?];[ ?]	Rank 1
P2			*c.[345*+*5G*>*A];[345*+*5G*>*A]*	p.[?];[?]	Rank 1
P7			c.[565C>T];[565C>T]	p.[R189*];[R189*]	Rank 1
ALG76			c.[626T>C];[626T>C]	p.[L209P];[L209P]	Rank 1
AKX44			c.[814C>T];[814C>T]	p.[R272*];[R272*]	Rank 1
AGL23			c.[1992delT];[1992delT]	p.[H665Tfs*675];[H665Tfs*675]	Rank 1
P13	***BBS5***	NM_152384.2	c.[149T>G];[149T>G]	p.[L50R];[L50R]	Rank 1
ALG5			c.[413G>A];[413G>A;]	p.[R138H];[R138H]	Rank 1
AIZ46	***MKKS***	NM_018848.3	c.[3G>A];[110A>G]	p.[M1I];[Y37C]	Rank 1, Rank 2
AIZ62			c.[571G>T];[724G>T]	p.[E191*];[A242S]	Rank 1, Rank 2
P10			*c.[1272*+*1G*>*A];[1272*+*1G*>*A]*	p.[?];[?]	Rank 1
ALB60	***BBS9***	NM_198428.2	c.[855del];[855del]	p.[W285*];[W285*]	Rank 1
ALS67	***BBS10***	NM_024685.3	c.[271_272insT];[728_731delAAGA]	p.[C91Lfs*95];[K243Ifs*257]	Rank 1, Rank 2
AMA70			c.[271_272insT];[1201G>T]	p.[C91Lfs*95];[G401*]	Rank 1, Rank 2
JSL			c.[285A>T];[2119-2120delGT]	p.[R95S];[V707*fs]	Rank 1, Rank 2
P8			c.[1181_1182insGCATTTAT];[1181_1182insGCATTTAT]	p.[S396Lfs*401];[S396Lfs*401]	Rank 1
AMR64			c.[1241T>C];[1241T>C]	p.[L414S];[L414S]	Rank 1
AKR68			c.[1241T>C];[1241T>C]	p.[L414S];[L414S]	Rank 2
ALP79	***BBS12***	NM_001178007.1	c.[865G>C];[205C>T(;)1859A>G]	p.[A289P];[L69F(;)Q620R]	Rank 1, Rank 2
ALB64	***ALMS1***	NM_015120.4	c.[1724C>G];[1724C>G]	p.[S575*];[S575*]	Rank 1
AIA84			c.[3340del];[3340del]	p.[E1112Rfs*1120];[E1112Rfs*1120]	Rank 1
ADC44			c.[7904insC];[7904insC]	p.[N2636Qfs*59];[N2636Qfs*59]	Rank 1
AKO26			c.[10879C>T];[10879C>T]	p.[R3627*];[R3627*]	Rank 1

**Notes.**

a The second mutation of the patient, a complete heterozygous deletion of exon 8 and 9 (c.(592-?)_(830+?)del) is a pathogenic CNV that cannot be ranked by VaRank.

b Parent of BBS patients, a single heterozygous mutations is expected.

c This validated mutation was filtered out in the “filteredVariants” results due to low sequencing quality (only 7 reads supported the variant) but ranked at the second position in the non-filtered results.

As mentioned in the original paper, one variant is always ranked in first position and represents a false positive described in *BBS2* as a third allele mutation according to the triallelic hypothesis (NM_031885.3:c.209G>A, rs4784677). It is flagged as pathogenic in dbSNP, but it is too frequent to be a fully penetrant mutation according to the observed frequency in the Exome Variant Server (EVS) (0.77%). Interestingly, using the barcode this variant is very easily filtered out since it is present in almost all patients in our cohort (31/32 samples).

### Intellectual disability (ID) dataset

Intellectual disability is a common neurodevelopmental disorder (∼2% of children and adolescents) (Ellison, Rosenfeld & Shaffer, 2013) that can be initiated by either environmental, genetic or multifactorial causes. The monogenic forms are very heterogeneous genetically, with several hundred genes identified so far. We present here the results of 203 patients affected with moderate to severe intellectual disability.

A first cohort of 107 patients was already analyzed by VaRank (Redin et al., 2014) and led to the identification of 25 causal mutations (Table 4A). A second cohort of 96 additional patients is reported here for which 12 causative mutations could be identified (Table 4B). The identified mutations were all validated by Sanger sequencing (we excluded pathogenic CNV). High-throughput sequencing data of protein-coding exons of 217 genes (first cohort) and 275 genes (second cohort) (either on the X-chromosome or associated to autosomal dominant or recessive forms of ID) were collected for the 25 positive known patients and the 96 additional ones. A total of 8,388 non redundant variants have been reannotated by VaRank using Alamut Batch version 1.1.11 and PolyPhen-2 v2.2.2, resulting on average into 1,129 (±189) private variants per sample. Each sample has been reanalyzed and a summary of the ranking results using the default filtration strategy for this dataset defined by the biologists (frequency in dbSNP or EVS >1% and presence in <3 samples) is available in Table 4. Among the 37 patients with causative mutations identified almost all were ranked first or within the top five among 1,129 variants on average demonstrating the usefulness of this strategy.

**Table 4:** Analysis of 203 patients with intellectual disability. (A) Mutations and ranking in the 25 positive patients from the 107 patients sequenced for 217 genes (Redin et al., 2014). (B) Mutations and ranking from 12 novel positive patients with ID identified in an additional cohort of 96 patients screened for 275 genes. Patients are sorted according to the mode of inheritance and the identified gene. Known mutations (from the literature) are highlighted in bold. Ranking into parenthesis highlights the ranking of the variations with a similar score. Mode of inheritance include: AD, autosomic dominant; AR, autosomic recessive; XL, X-linked; XLD, dominant on the X chromosome.

(A)							
Patient#	Sex	Gene	Chromosome	Mode of inheritance	Mutation (cDNA)	Mutation (protein)	Ranking
APN-58	M	*DYRK1A*	21	AD	c.[613C>T];[=]	p.[R205*];[=]	Rank 2
APN-87	M				c.[621_624delinsGAA];[=]	p.[E208Nfs*3];[=]	Rank 1
APN-63	M	*GRIN1*	9	AD	c.[1733C>G];[=]	p.[P578R];[=]	Rank 1 (2)
APN-14	M	*MED13L*	12	AD	c.[6118_6125del];[=]	p.[G2040Nfs*32];[=]	Rank 1 (2)
APN-46	M	*RAI1*	17	AD	c.[2332_2336del];[=]	p.[G778Efs*7];[=]	Rank 1
APN-122	F	*SHANK3*	22	AD	c.[2955_2970dup];[=]	p.[P992Rfs*325];[=]	Rank 1
APN-38	M	*SLC2A1*	1	AD	c.[724C>T];[=]	p.[E242*];[=]	Rank 2
APN-139	M	*SYNGAP1*	6	AD	c.*[3583-6G>A]*;[=]	p.[?];[?]	Rank 1
APN-41	M	*TCF4*	18	AD	c.[514_517del];[=]	p.[K172Ffs*61];[=]	Rank 1
APN-117	F				c.[520C>T];[=]	p.[R174*];[=]	Rank 1
APN-138	M	*ATRX*	X	XL	**c.[109C>T];[0]**	p.[R37*];[0]	Rank 1 (2)
APN-137	M	*CUL4B*	X	XL	c.[811_812del];[0]	p.[E271Aspfs*11];[0]	Rank 1 (2)
APN-42	M	*DMD*	X	XL	c.[10889del];[0]	p.[R3630Efs*27];[0]	Rank 1
APN-113	M	*HCFC1*	X	XL	**c.[218C>T];[0]**	**p.[A73V];[0]**	Rank 1
APN-82	M	*IL1RAPL1*	X	XL	c.[894_903del];[0]	p.[W299Tfs*18];[0]	Rank 1
APN-68	M	*IQSEC2*	X	XL	c.[3097C>T];[0]	p.[E1033*];[0]	Rank 1 (2)
APN-34	M	*KDM5C*	X	XL	c.[2152G>C];[0]	p.[A718P];[0]	Rank 1
APN-135	M				c.[1296dup];[0]	p.[E433*];[0]	Rank 1
APN-16	M	*MAOA*	X	XL	c.[797_798delinsTT];[0]	p.[C266F];[0]	Rank 1
APN-130	F	*MECP2*	X	XLD	**c.[952C>T];[**=**]**	**p.[R318C];[**=**]**	Rank 2^a^
APN-142	F				**c.[538C>T];[**=**]**	p.[R180*];[=**]**	Rank 1^a^
APN-105	M	*PHF8*	X	XL	c.[1249+5G>C];[0]	p.[Y406Ffs*24];[0]	Rank 4
APN-43	M	*SLC9A6*	X	XL	c.*[526-9_526-5del]*;[0]	p.[?];[0]	Rank 1
APN-110	M	*SLC16A2*	X	XL	**c.[1412T>C];[0]**	**p.[L471P];[0]**	Rank 1

(B)							
Patient#	Sex	Gene	Chromosome	Mode of inheritance	Mutation (cDNA)	Mutation (protein)	Ranking
APN-206	F	*ANKRD11*	16	AD	c.[2904del];[=]	p.[Glu969Argfs*8];[=]	Rank 1 (2)
APN-237	F	*DYRK1A*	16	AD	c.[1205dup];[=]	p.[Arg404Thrfs*10];[=]	Rank 1 (2)
APN-176	F	*FOXG1*	14	AD	c.[755G>T];[=]	p.[Gly252Val];[=]	Rank 2 (3)
APN-188	F	*MBD5*	2	AD	c.[3949del];[=]	p.[Gln1317Serfs*48];[=]	Rank 1
APN-211	M	*MED13L*	12	AD	c.[2340_2343de];[=]	p.[Thr781Metfs*19];[=]	Rank 1 (2)
APN-200	M	*SYNGAP1*	6	AD	c.[1717C>T];[=]	p.[Arg573Trp];[=]	Rank 2
APN-226	F	*TBR1*	3	AD	c.[713_719del];[=]	p.[Ser238Thrfs*17];[=]	Rank 2 (2)
APN-149	M	*TCF4*	18	AD	c.*[990G>A]*;[=]	p.[?];[=]	Rank 1
APN-214	M				c.[1726C>T];[=]	p.[Arg576*];[=]	Rank 2
APN-210	F				c.**[1733G>A]**;[=]	p.**[Arg578His]**;[=]	Rank 2 (2)^a^
APN-209	F	*ASPM*	1	AR	c.[2967G>A];[ 6920_6921del]	p.[Trp989*];[Gln2307Leufs*10]	Rank 1, Rank 2
APN-162	M	*KIAA2022*	X	XL	c.[2725del];[0]	p.[Ala909Profs*13];[0]	Rank 1

**Notes.**

a Known mutation not annotated as pathogenic in dbSNP.

As an example, in a boy with autism spectrum disorder, attention deficit and autoaggressive behavior, one mutation (c.797_798delinsTT, p.C266F) has been observed in the *MAOA* gene. A total of 688 variants have been annotated by VaRank and the mutation described for this patient could be ranked without filtering at position 8. When the usual filters are applied (frequency in dbSNP or EVS >1%), the mutation is ranked at the first position in the “filteredVariants.rankingByVar.txt” file. This result was the first mutation report in the *MAOA* gene since 20 years (Piton et al., 2014).

### Whole Exome Sequencing (WES) dataset

WES of a single patient with a clear BBS phenotype from a consanguineous Italian family (Scheidecker et al., 2014) revealed for the first time mutations in the *BBIP1* gene counting since as the 18th BBS gene. A total of 50,569 non redundant variants have been annotated by VaRank using Alamut Batch version 1.1.11 and PolyPhen-2 v2.2.2.

The nonsense mutation c.173T>G (p.Leu58*) in *BBIP1* has been ranked at the 50th position/50569 in the “allVariants.rankingByVar” file. Applying the default filtration strategy (i.e., frequency in dbSNP or EVS >1% and sequence quality filters) changed the ranking to the 6th position/4,908 (“filteredVariants.rankingByVar” file). This exome sequencing dataset was analyzed together with 29 other exomes from unrelated patients and pathologies for a BBS patient for which we could further filter variations using for example the barcode. Given that the most frequent known disease causing mutation in BBS is the c.1169T>G (p.M390R) mutation in *BBS1* (Mykytyn et al., 2002), found in EVS at the frequency of 26/12,694 (0.2%) at the heterozygous state, we used the barcode statistics to further reduce the total number of variations. Being very tolerant, we kept variants present less than four times at the heterozygous state or two times at the homozygous state out of 29 samples. The final ranking placed the mutation at the forth position (and first homozygous) out of 3,493 remaining variants.

**Figure 5:**
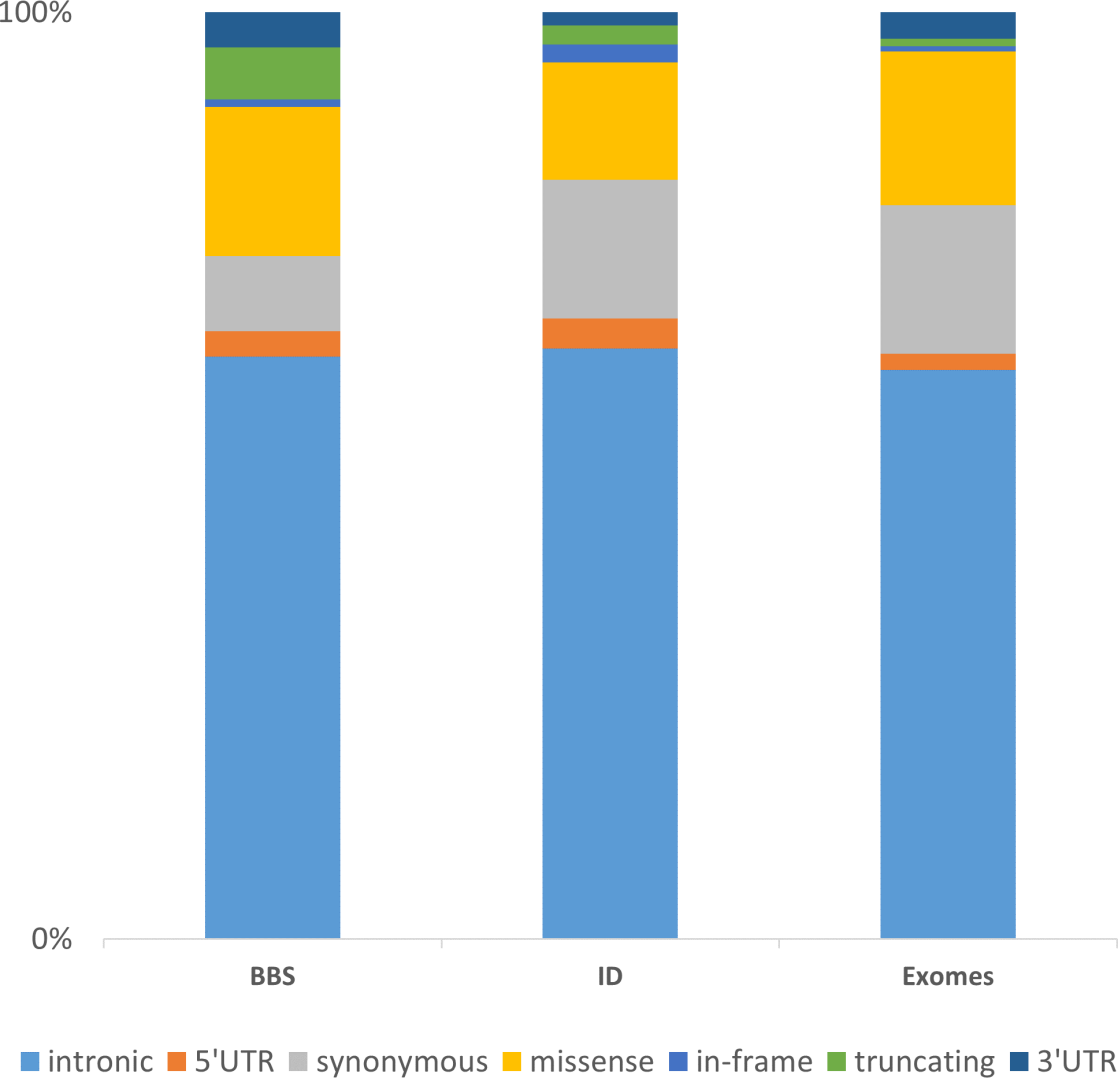
Representation of the non-redundant variations by functional type in 3 datasets. The chart is built upon the Intellectual disability (ID) and Bardet-Biedl Syndrome (BBS) (consolidated from 188 patients addressed for BBS) datasets discussed in the Results section together with an enhanced exome dataset (35 exomes). The “truncating” category corresponds to frameshift, nonsense, stoploss and startloss.

The integration of these three datasets of increasing complexity (BBS with 30 genes consolidated on 188 patients, ID with more than 200 genes in 121 patients and the WES data consolidated for 35 samples) highlights major directions for further developments. Considering the distribution of the non-redundant variation by functional category, one can first observe, despite a different gene composition, that the distribution of the categories is similar among the datasets (Fig. 5). The vast majority of the identified and annotated variations are either intronic or synonymous. Those categories contain known variations in dbSNP (∼85% have an rs#) and are either polymorphisms or rare variant. Thus they are often considered as non-pathogenic. Nevertheless, some of these could potentially affect the correct splicing of the closest gene. Although efforts are being taken to enhance the predictions on splicing especially for the consensus sequences (Houdayer et al., 2012; Jian, Boerwinkle & Liu, 2014), little is done in more distant regions including enhancers sequences, branch point or even promoter regions. The 5’ and 3’ UTRs are also sources of variations that are often overlooked but contain important functional signals such as miRNA binding sites and polyadenylation signal (Chatterjee & Pal, 2009). Missenses are one of the major variations sources and also one of the more difficult to interpret. There is a high number of predictions methods and tools available aiming at predicting the pathogenicity of this category of variant but there is still improvement to be made (Flanagan, Patch & Ellard, 2010; Hicks et al., 2011; Tavtigian et al., 2008). Recent approaches aimed at combining knowledge based information such as structural information for missense variants (Luu et al., 2012) or focusing only on orthologous sequences (Wong & Zhang, 2014). Other interesting annotation sources might be included as the assessment of non-frameshift indels (Bermejo-Das-Neves et al., 2014; Hu & Ng, 2012) and the consideration of CNVs to improve the decision-making. Those issues will be amplified using whole genome sequencing (WGS).

## Conclusion

The rationale behind VaRank is to provide a simple yet powerful tool for biologists and researchers aiming to discover new human disease causing variants (i.e., mutations) from DNA sequencing projects. VaRank is a comprehensive workflow for annotating and ranking SNVs and indels that aims at collecting the major annotation sources. The program is currently compatible with two annotation software namely Alamut Batch and SnpEff. This could further expanded to other annotation solutions such as Annovar (Wang, Li & Hakonarson, 2010) or VEP (McLaren et al., 2010). It stands out from other solutions by being able to provide a summarized overview of the presence/absence status of each variant within all patients (e.g., barcode and family barcode) and allowing users to easily test several disease transmission hypothesis (recessive, dominant…). The barcode counts can serve as an internal frequency database in order to filter out known and unknown frequent variants together with annotation errors and recurrent sequencing errors. Moreover, a specific ranking for each gene is particularly appropriate in the case of recessive diseases. Finally, as a command line tool it can easily be integrated into existing bioinformatics pipelines and accelerate identification of causal variants. The manual and a tutorial together with changelogs and various use cases are available via our dedicated website at http://www.lbgi.fr/VaRank.

## Supplemental Information

10.7717/peerj.796/supp-1 Supplemental Information 1 VaRank’s source code

10.7717/peerj.796/supp-2 Supplemental Information 2 VaRank’s readme

10.7717/peerj.796/supp-3 Supplemental Information 3 VaRank’s tutorial
