# Supplementary material for: VaRank: a simple and powerful tool for ranking genetic variants
Source: PeerJ. 2015 Mar 3;3:e796. doi: 10.7717/peerj.796 (PMC4358652; doi:10.7717/peerj.796)
Supplement: Supplemental Information 1 [file peerj-03-796-s001.gz › VaRank_1.2.1/README.VaRank_1.2.pdf]

VaRank Manual

Version 1.2
VaRank is a program for genetic Variant Ranking from NGS data

Copyright (C) 2015 GEOFFROY Véronique, MULLER Jean

Please feel free to contact us for any suggestions or bug reports
email: veronique.geoffroy@inserm.fr; jeanmuller@unistra.fr

==
TABLE OF CONTENTS
==

1. INTRODUCTION

2. INSTALLATION/REQUIREMENTS

3. INPUT

4. OUTPUT

5. SCORING

6. USAGE / OPTIONS

7. ANNOTATION COLUMNS

8. FAQ

==

1. INTRODUCTION
===============

VaRank is a program designed for variant ranking from next generation sequencing data. It provides a comprehensive workflow for annotating and ranking SNVs and indels.
Four modules create the strength of this workflow:
(i) Integration of the sequencing data: variant call quality summary (total and variant depth of coverage, phred like information), to filter out false positive calls.
(ii) Alamut Batch or SnpEff annotations, to integrate genetic and predictive information (functional impact, putative effects in the protein coding regions, population frequency...) from different sources, using HGVS nomenclature.
(iii) Barcode representing the presence/absence of variants (with homozygote/heterozygote status), to search for recurrence between families or group of individuals.
(iv) Prioritization score, to rank variants according to their predicted pathogenic status.

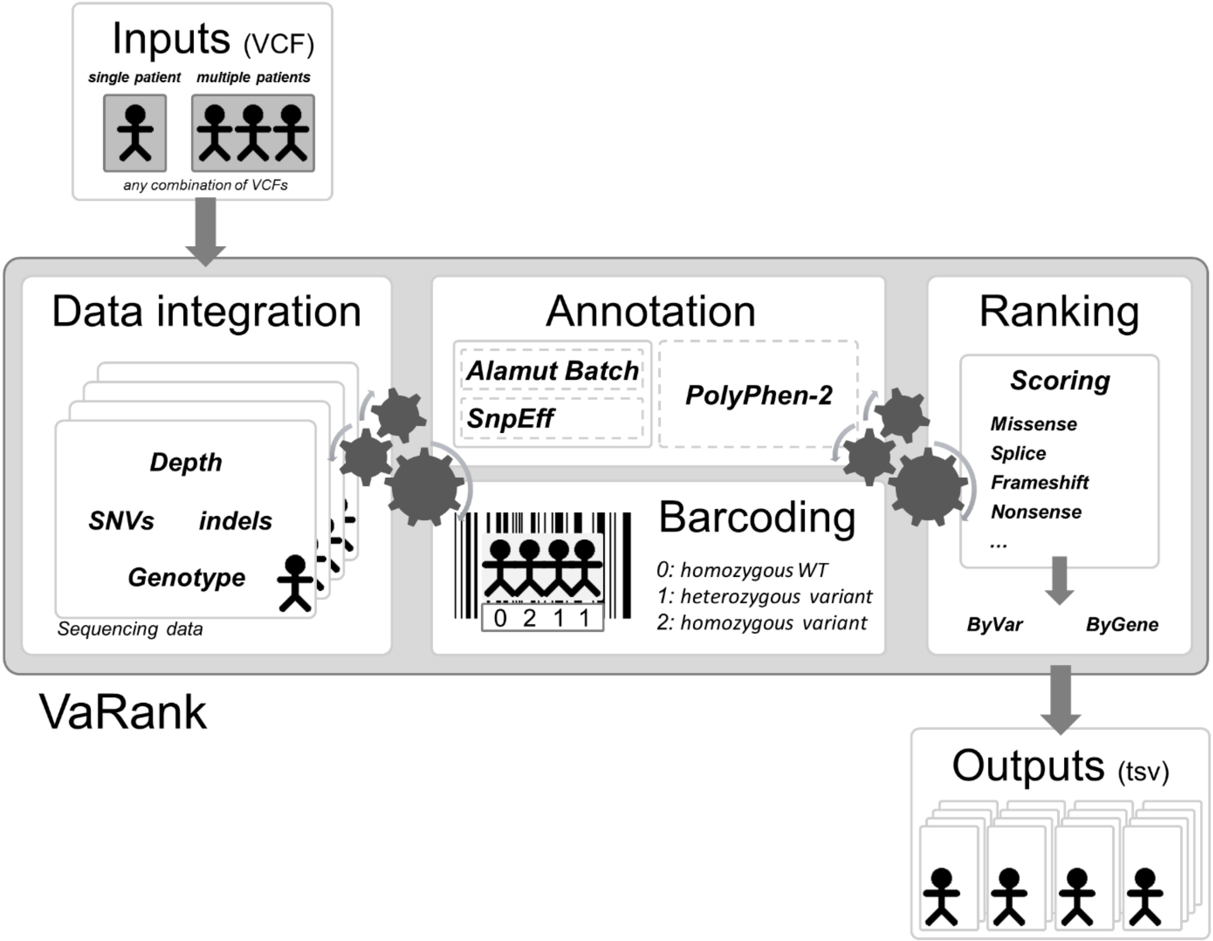

2. INSTALLATION/REQUIREMENTS
============================

The VaRank program is written in the Tcl/Tk language. Modern Unix systems have this scripting language already installed (otherwise it can be downloaded from http://www.tcl.tk/).This tool is composed of different other programs and databases:

- VaRank sources can be downloaded from http://www.lbgi.fr/VaRank under the GNU GPL license.

- The annotation engine from either:
    - Alamut Batch developed and commercialized by Interactive Biosoftware (Rouen, France). If you do not own a license, a 30-day free trial can be requested here (http://www.interactive-biosoftware.com/request-trial-alamut/).
    - SnpEff and SnpSift developed by Pablo Cingolani (http://snpeff.sourceforge.net/)

Optional:
- PolyPhen-2 (PPH2) provides prediction of functional effects of human nsSNPs (Adzhubei IA *et al* Nat. Methods 2010). It needs to be locally installed to be used. You can freely download it from http://genetics.bwh.harvard.edu/pph2/dokuwiki/downloads

- Protein databases can be used to connect to PPH2. UniProt and RefSeq can respectively be downloaded from the proposed URL or procedures and should placed in the "Databases" directory.
    - UniProt:

The human reference protein file can be downloaded from the following command:

```
wget
ftp://ftp.uniprot.org/pub/databases/uniprot/current_release/knowledgebase/proteomes/H
UMAN.fasta.gz
```

- RefSeq

Since the 25/11/2014, the organization of the RefSeq repository has changed. The human.protein.faa.gz file which contained the whole human protein sequences is now splitted into multiple files. Please run these commands to download and prepare the data for VaRank and copy them in the:

```
wget -rnd -A 'human.*.protein.faa.gz' ftp://ftp.ncbi.nlm.nih.gov/refseq/H_sapiens/mRNA_Prot/
foreach file (*.protein.faa.gz)
echo "reading $file"
cat $file >> human.protein.faa.gz
chmod 755 $file
rm $file
end
mv human.protein.faa.gz $VARANK/Databases/
```

The protein databases files help VaRank to extract the protein sequences and 1/ check the amino acid change to be tested and 2/ submit the protein sequence to PPH2 if no accession are precomputed.

VaRank sources installation

The source .tar.gz should be extracted and uncompressed to any directory.

```
mkdir VaRank
cd VaRank
wget http://www.lbgi.fr/VaRank/Sources/VaRank_latest_core.tar.gz
tar -xvf VaRank_latest_core.tar.gz
gunzip VaRank_latest_core.tar
```

The installation requires simply to set the following environment variables:
- $VARANK : VaRank installation directory

Depending on the selected annotation engine:
- $ALAMUT : Alamut Batch installation directory
- $SNPEFF : SnpEff and SnpSift installation directory

Alamut Batch installation

For the installation of Alamut Batch, we recommend the use of the standalone version which is very handy to install with a single tar.gz file and a single database file, and more efficient for the annotation. The first use of Alamut Batch requires the end user license agreement, we recommend to do it right after the installation.

SnpEff installation

The installation of SnpEff and SnpSift is also well described on its website. Apart from the programs additional databases should be downloaded (the human reference genome, dbSNP, dbNFSP, phastCons). Make sure you are running the required java version (i.e. SnpEff 4.1 requires Java 1.7). You should check the following sections and download the corresponding files:

- <u>SnpEff and SnfSift</u>

```
cd /path/to/SnpEff/dir/
wget http://sourceforge.net/projects/snpeff/files/snpEff_latest_core.zip
unzip snpEff_latest_core.zip
cd SnpEff

#Define the SNPEFF environment variable
setenv SNPEFF /path/to/SnpEff/dir/snpEff/
mkdir Test_VaRank
#Copy one vcf example file from the $SNPEFF/examples directory
cp examples/test.vcf VaRank

#To check if the SnpEff is running properly
#In this example the '-v GRCh37.75' will automatically download the human reference
#genome if not yet downloaded. You can change the value to match another version.
java -Xmx4g -jar $SNPEFF/snpEff.jar eff -c $SNPEFF/snpEff.config -v GRCh37.75
$SNPEFF/Test4VaRank/test.vcf > $SNPEFF/Test4VaRank/file.eff.vcf
#No error message validate this step, one can still have a look at the output file

#To check if the SnpSift is running properly
java -Xmx4g -jar $SNPEFF/SnpSift.jar varType $SNPEFF/Test4VaRank/file.eff.vcf >
$SNPEFF/Test4VaRank/file.eff.varType.vcf
#No error message validate this step, one can still have a look at the output file
```

- <u>Additional databases</u>

The followings commands and links will guide you trhough the installation and proper installation of SnpEff for the use with VaRank.
http://snpeff.sourceforge.net/SnpEff_manual.html
http://snpeff.sourceforge.net/SnpSift.html#VariantType

To install **dbSNP** (http://snpeff.sourceforge.net/SnpSift.html#annotate)

```
cd $SNPEFF
mkdir -p db/dbSNP
cd db/dbSNP
#To check if SnpSift is properly using dbNSFP data
wget ftp://ftp.ncbi.nih.gov/snp/organisms/human_9606/VCF/00-All.vcf.gz
gunzip 00-All.vcf.gz
#To check if SnpSift is properly using dbSNP data
java -Xmx4g -jar $SNPEFF/SnpSift.jar annotate $SNPEFF/db/dbSNP/00-All.vcf
$SNPEFF/Test4VaRank/file.eff.varType.vcf >
$SNPEFF/Test4VaRank/file.eff.varType.dnsnp.vcf
#No error message validate this step, one can still have a look at the output file
```

To install **dbNSFP** (http://snpeff.sourceforge.net/SnpSift.html#dbNSFP)

```
cd $SNPEFF
mkdir -p db/dbNSFP
cd db/dbNSFP
wget http://sourceforge.net/projects/snpeff/files/databases/dbNSFP/dbNSFP2.4.txt.gz
wget
http://sourceforge.net/projects/snpeff/files/databases/dbNSFP/dbNSFP2.4.txt.gz.tbi

#To check if SnpSift is properly using dbNSFP data
java -Xmx4g -jar $SNPEFF/SnpSift.jar dbnsfp -db $SNPEFF/db/dbNSFP/dbNSFP2.4.txt.gz
$SNPEFF/Test4VaRank/file.eff.varType.vcf >
$SNPEFF/Test4VaRank/file.eff.varType.dbnsfp.vcf
#No error message validate this step, one can still have a look at the output file
```

To install **phastCons** (http://snpeff.sourceforge.net/SnpSift.html#phastCons)

```
cd $SNPEFF
mkdir -p db/phastCons/
cd db/phastCons
foreach c (1 2 3 4 5 6 7 8 9 10 11 12 13 14 15 16 17 18 19 20 21 22 M X Y)
wget
http://hgdownload.soe.ucsc.edu/goldenPath/hg19/phastCons100way/hg19.100way.phastCons/
chr$c.phastCons100way.wigFix.gz
end
wget http://snpeff.sourceforge.net/genome.fai
cd $SNPEFF
#To check if SnpSift is properly using phastCons data
java -Xmx4g -jar $SNPEFF/SnpSift.jar phastCons $SNPEFF/db/phastCons
$SNPEFF/Test4VaRank/file.eff.varType.vcf >
$SNPEFF/Test4VaRank/file.eff.varType.phastCons.vcf
#No error message validate this step, one can still have a look at the output file
```

It is to notice that in order to overcome, the fact that the annotation of some deletions by SnpEff is not clear enough for multiple alleles at the same position, variations with multiple alleles are split into multiple variant/lines while creating the non-redundant input vcf files. During this process the genotype are stored in memory but modified in the vcf files to “0/1” by default. These specific vcf files should then not be used for any other purpose (VcfDirectory/SnpEff/Input).

While running VaRank with SnpEff some parameters should be used including: -snpeffHumanDB, -dbSNP, -dbNSFP and –phastConsDB (see section 6 for more details).

The following environment variable is optional:
- $PPH : PolyPhen-2 installation directory

By default the VaRank installation directory looks like this:

```
VaRank                         #The program installation directory
 |
 |----- bin/                   #Where an alias is set to the main .tcl script
 |
 |----- DataBases/             #Where to store the UniProt and RefSeq fasta files
 |      |----- SnpEff/         #Contains an example to check SnpEff execution
 |
 |----- sources/               #Where the .tcl files are stored
 |
 |----- configfile             #an ex of configfile that can be copied to any analysis director
 |                             #for modification purpose
 |
 |----- changeLogs.txt         #description of VaRank changes
 |
 |----- License.txt            #GNU GPL license
 |
 |----- README.VaRank.*.pdf    #This file
```

Make sure the program find correctly the Tcl interpreter, by default the best way to make a Tcl script executable is to put the following as the first line of the main script (which is already done in VaRank-main.tcl):

```
#!/usr/bin/env tclsh
```

But it can be changed to any other path like:
```
#!/usr/local/ActiveTcl/bin tclsh
```

Typically, you can create an alias of the main Tcl script “sources/VaRank-main.tcl” for example to “VaRank”, place it in the “/bin” directory” (this is done be default already) and add the path to this in your $PATH.

3. INPUT
=======

VaRank supports the commonly used VCF (Variant Call Format, https://github.com/samtools/hts-specs) input format for variants analysis that allows the program to be easily integrated into NGS bioinformatics analysis pipelines. Gzip-format VCF files are supported.

VaRank takes also several argument as options to the command line that are detailed in section 6 (“USAGE / OPTIONS”). The different arguments can be passed either on the command line or using a specific file named "configfile" that needs to be put in the same directory as the input VCF files. An example of configfile is provided in the VaRank installation directory.

a. Family Barcode

The barcode in VaRank allows a quick overview of the presence/absence status of each variant and their zygosity status within the analyzed individuals (“0” representing homozygous wild type, “1” heterozygous and “2” homozygous for the variant, see the figure below Panel A). Panel B displays 3 variants example and 32 patients analyzed together.

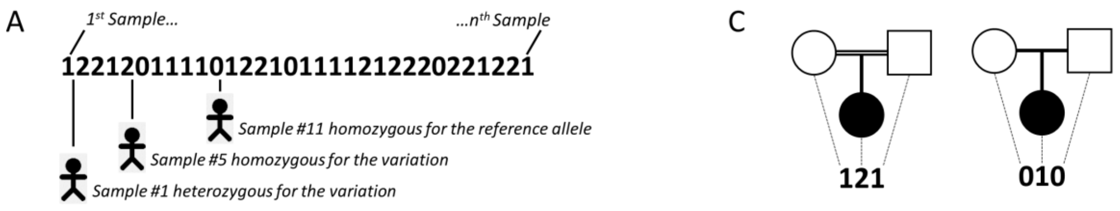

B

Gene	Chr	Start	Ref	Mut	Zygosity	TotalRead Depth	VarRead Depth	cNomen	pNomen	familyBarcode	Barcode	#Hom	#Het	#Allele	#Sample
BBS2	16	56548501	C	T	hom	142	142	c.209G>A	p.=	**222**	**22222222202222222222222222222222**	31	0	62	32
ALMS1	2	73716993	-	C	hom	143	126	c.7911dup	p.Asn2638Glnfs*24	**121**	**00000000000000000000000000200000**	1	0	2	32
TTC21B	2	166797646	C	T	het	144	80	c.601G>A	p.Val201Met	**011**	**12211011112122101111212220121221**	12	17	41	32

Together with the main barcode describing all the patients analyzed together in one VaRank run, one can define a second barcode. This second barcode named “*familyBarcode*” can be configured by the user to group selected samples (e.g. trios where affected child and parents could be specifically grouped together). This can be configured in the configfile by simply grouping sample names together. As an example, 2 families where the fam1 corresponds to a trio sequencing (proband and parents, see Figure Panel C) and fam2 with 2 affected child.

fam1: Sample1 Sample2 Sample3
fam2: Sample4 Sample5

Grouping sample names together allows also to follow the same naming convention for the files with the same prefix (fam1_ for all family members).

b. External Gene annotation

In order to further enrich the annotation for each variant and each gene, VaRank can integrate (using the option -extann) external annotations imported from a tab separated values file into the output files (gzip files are supported). The file format is easy and should look like this (1st line is a header including a column entitled "Gene" that should be the 1st column too). The following example has been set to provide annotation for the gene including the transmission mode of the gene (here AR means “autosomic recessive”), the number of missense and truncating mutations reported as well as the OMIM identifier.

Gene	Transmission	#Missense	#Truncating	Omim
ACY1	AR	4	2	104620
ADSL	AR	7	1	608222

4. OUTPUT
=========
VaRank provides 4 .tsv (TAB separated values) output files divided into 2 categories:

-Files named with "ByVar" contains variations sorted from the most to the least pathogenic (according to the VaRank score)
-Files named with "ByGene" contains variations classified by gene ("ByGene") where the list is sorted using the gene as a proxy to the score. Each gene is scored according to most pathogenic variant (homozygous) or the first two most pathogenic variants. In order to make sure that no variants are missed all gene variation are reported also below the variant(s) used to score the gene. This file is more suitable when dealing with a recessive mode of inheritance.
It is to notice that given the focus on genes in those output files, variants that could be attributed to several genes are duplicated and associated to each gene individually.

A part from these 2 categories, each file is also available in 2 versions:

-Raw file ("allVariants") with no variants filtered out.
-Already prefiltered files ("filteredVariants") with variants filtered out using the following criteria:

The default filters remove variants:

-with a total depth of coverage <= 10x
-with a supporting reads count <= 10x
-with a percent of supporting reads <= 15%
-with validated annotation in the dbSNP database (i.e. at least with 2 evidences) that are not pathogenic (from the ClinicalSignificance field in dbSNP)
-with an allele frequency > 1% (extracted from the dbSNP database or the Exome Variant Server)

The “filtered” files can be considered as very stringent filtering step to ensure a very quick first analysis of the data. Users can always adapt the options to make fit his situation.

The output organization can be described as follows:

```
VcfDirectory
 |
 |----- configfile                                #if present can be used to define sample group and set
 |                                                #options of VaRank
 |
 |----- *InputFile.vcf/.vcf.gz                    #Input files
 |
 |-- Alamut/                                      #Contains all Alamut Batch related files
 |   |-- AlamutInputFile_all.txt                  #Alamut input file generated from the vcf(s) files
 |   |-- AlamutAnnotations_all.txt                #Alamut output file with annotated variants
 |   |-- AlamutUnnanotated_all.txt                #Alamut output file with unannotated variants
 |   |-- AlamutOutput_all.txt                     #Alamut log file
 |
 |-- SnpEff/                                             #Contains all SnpEff and SnpSift related files
 |   |-- Input/                                          #Contains all SnpEff and SnpSift related files
 |       |-- *.vcf                                       #Non redundant variant input vcf files
 |   |-- Output/                                         #Contains all SnpEff and SnpSift related files
 |       |-- *.varType.vcf.log                           #SnpSift varType annotation log
 |       |-- *.varType.dbsnp.vcf.log                     #SnpSift dbSNP annotation log
 |       |-- *.varType.dbsnp.dbnsfp.vcf.log              #SnpSift dbsnfp annotation log
 |       |-- *.varType.dbsnp.dbnsfp.phastCons.vcf.log    #Log file for the final annotation step
 |       |-- *.varType.dbsnp.dbnsfp.phastCons.vcf        #Final annotation file
 |
 |-- PPH2/                                        #(option) Contains all PolyPhen-2 related files
 |   |-- PPH2input_all.txt                        #PPH2 input file
 |   |-- PPH2features_all.txt                     #PPH2 output file
 |   |-- PPH2humvar_all.txt                       #PPH2 output file
 |   |-- PPH2errors_all.txt                       #PPH2 log file
 |
 |----- fam#_SampleName_allVariants.rankingByVar.tsv
 |----- fam#_SampleName_filteredVariants.rankingByVar.tsv
 |
 |----- fam#_SampleName_allVariants.rankingByGene.tsv
 |----- fam#_SampleName_filteredVariants.rankingByGene.tsv
 |
 |----- fam#_SampleName_statistics.tsv            #Short counts report (e.g. homozygous, heterozygous
 |                                                #and total counts) for each of the variant categories
 |
 |----- SNV_global_statistics.tsv                 #Contains the same counts as defined for each patient
 |                                                #but for the whole analyzed cohort
```

a. VariantID

The output files contains a columns named *VariantID* which is a variation identifier meant to be unique. The format is described as follows:

[#chr]_[genomicposition]_[RefBase]_[VarBase]

[RefBase] being the nucleotide sequence in the reference genome
[VarBase] being the alternate nucleotide sequence.

Ex1: 16_56548501_C_T describes the change of C to T on chromosome 16 at position 56548501.

In order to optimize the description of this identifier for larger indels, the [RefBase] and [VarBase] values are restricted to 50bp by default.
Ex2: 21_9448722_330bp_- describes the deletion on chromosome 21 of 330pb.

In case of redundancy (e.g. insertion of different sequences at the same position of the same size) in order to keep non redundant identifiers a versioning is applied.
Ex3: 21_9448722_-_89bp and 21_9448722_-_89bp.1 correspond to the insertion of 2 different sequences of the same length on chromosome 21.

b. Absence of annotations

It is to notice that when no annotation is available for a specific column, the empty value is set to "NA". Exception is made for several numerical columns (including *rsMAF, espEAMAF, espAAMAF, espAllMAF*) where "-1" is used that allows the user to further filter information without losing data.

5. SCORING
==========
VaRank uses the variation type (i.e. substitution, deletion, insertion, duplication) and the coding effect to score. The VaRank scoring is categorized from the most likely to the less likely pathogenic state as follows (score into parenthesis): known mutation (110), nonsense (100), frameshift (100), essential splice site (2 first bases before and after the exon) (90), start loss (80), stop loss (80), intron-exon boundary (donor site is -3 to +6, acceptor site -12 to +2) (70), missense (50), in-frame (40), deep intronic changes (25) and synonymous coding (10). Each category is further described in the USAGE/OPTIONS section and each score can be changed.

Each specific variant score is further adjusted using additional information. For this, variants are assessed at the genomic level (phastCons) and at the protein level (SIFT and if installed PolyPhen-2), and an adjustment score (0 or +5) is added to the relevant category. The adjustment score can be changed by the user.
To ensure the best use of SIFT predictions, the deleterious status is only taken if the SIFT median value is comprised between [2.75-3.5].

Scores in bold reflect score values after the adjustment score is applied. 1Each variant score is adjusted (+5) if high conservation at the genomic level is observed (phastCons cutoff >0.95). 2Missense scores are adjusted (+5) for each deleterious prediction (SIFT and/or PPH2).

Variant Category	Option name	VaRank Score	Definitions
**Known mutation**	*S_Known*	110	Known mutation as annotated by HGMD and/or dbSNP (rsClinicalSignificance="pathogenic/probable-pathogenic")
**Nonsense**	*S_Nonsense*[1]	100, **105**	A single-base substitution in DNA resulting in a STOP codon (TGA, TAA or TAG).
**Frameshift**	*S_Fs*	100	Exonic insertion/deletion of a non-multiple of 3bp resulting often in a premature stop in the reading frame of the gene.
**Essential splice site**	*S_EssentialSplice*[1]	90, **95**	Variation in one of the canonical splice sites resulting in a significant effect on splicing.
**Start loss**	*S_StartLoss*[1]	80, **85**	Variation leading to the loss of the initiation codon (Met).
**Stop loss**	*S_StopLoss*[1]	80, **85**	Variation leading to the loss of the STOP codon.
**Intron-exon boundary**	*S_CloseSplice*[1]	70, **75**	Variation outside of the canonical splice sites (donor site is -3 to +6, acceptor site is -12 to +2).
**Missense**	*S_Missense*[1,2]	50, **55, 60, 65**	A single-base substitution in DNA not resulting in a change in the amino acid.
**Indel in-frame**	*S_Inframe*	40	Exonic insertion/deletion of a multiple of 3bp.
**Deep intron-exon boundary**	*S_DeepSplice*[1]	25, **30**	Intronic variation resulting in a significant effect on splicing.
**Synonymous coding**	*S_Synonymous*[1]	10, **15**	A single-base substitution in DNA not resulting in a change in the amino acid.

6. USAGE / OPTIONS

==================

A complete tutorial together with examples are available on the website to further describe the use of VaRank. To run VaRank, the default command line is the following:

```
$VARANK/bin/VaRank -vcfdir '/Path/To/The/Directory/Containing/vcf/files' >& log.log &
```

The command line can be completed by the list of options described below or modified in the configfile. To show the options simply type:

```
$VARANK/bin/VaRank -help or $VARANK/bin/VaRank
```

OPTIONS:

-help	More information on the arguments
-vcfDir	Path of your study directory containing your vcf input file
-vcfInfo	To extract the info column from the .vcf file and insert the data in the output file (last columns).<br>Range values: yes or no (default)
-rsfromvcf	To extract the rsID and validation status from the .vcf file and insert this in the output file.<br>Range values: yes or no (default)
-nowebsearch	To allow or not the access to the web for downloading the fasta sequences for missed proteins in UniProt and/or RefSeq (only suitable when used with PolyPhen-2). It is to notice that the search can be very time consuming since getting sequences one by one.<br>Range values: yes (default) or no
-Homstatus	To force the determination of the homozygous or heterozygous state of one variation. If set to yes it will use the Homcutoff value to decide.<br>Range values: yes or no (default)
-Homcutoff	To determine the homozygous or heterozygous state of one variation. If set to some value it will force to reconsider the data provided.<br>Range values: [0,100] default: 80 (active only if Homstatus=yes or when no status is given)
-MEScutoff	MaxEntScan cutoff, to determine the impact of the variant on splicing. Expressed as the % difference between the variant and the WT score.<br>Range values: [-100,0], default: -15
-SSFcutoff	Splice Site Finder cutoff, to determine the impact of the variant on splicing. Expressed as the % difference between the variant and the WT score.<br>Range values: [-100,0], default: -5
-NNScutoff	NNSplice cutoff, to determine the impact of the variant on splicing. Expressed as the % difference between the variant and the WT score.<br>Range values: [-100,0], default: -10
-phastConsCutoff	To determine when a genomic position is conserved or not. Above the cutoff is considered as conserved.<br>Range values: [0,1], default: 0.95
-readFilter	Minimum number of reads for the variants<br>Range values: [0,-], default: 10
-depthFilter	Minimum depth for the variants<br>Range values: [0,-], default: 10
-readPercentFilter	Minimum percent of variant reads for considering a variant<br>Range values: [0,100], default: 15
-freqFilter	Filtering variants based on their MAF in the SNV databases (dbsnp and EVS)<br>Range values: [0.0,1.0], default: 0.01

-rsFilter	Filtering variants on the SNP informations<br>Values: removeNonPathoRS (remove variants without "probable-pathogenic" or "pathogenic" annotation, see clinical significance field in dbSNP website. Filtering only for variants with at least 2 validations.)<br>none = keep all variants, no filtering on rsID<br>Default: removeNonPathoRS
-extann	Tab separated file containing annotation to add to the final output files. Restrictions for the format are: 1st line is a header, 1st column is the gene name<br>Typical use would be a gene file containing specific annotations such as transmission mode, disease, expression...
-metrics	Changing numerical values from frequencies to us or fr metrics (e.g. 0.2 or 0,2)<br>Range values: us (default) or fr
-DB	Changes the directory where the UniProt and Refseq files are stored (optional, only use if PPH2 is installed)<br>Ex: $VARANK/Databases (default)
-alamutHumanDB	Alamut Batch specific option to select the reference human genome version
-javaPath:	To make sure the java path is set up properly you can enter it here
-snpeffHumanDB	SnpEff specific option to select the reference human genome version<br>Ex: "GRCh37.75"
-dbSNP	SnpEff specific option to describe the full path to the location of the dbSNP vcf file used by SnpSift<br>Ex: "$SNPEFF/db/dbSNP.2015-01-09_00-All.vcf"
-dbNSFP	SnpEff specific option to describe the full path to the location of the dbNSFP vcf file used by SnpSift<br>Ex: "$SNPEFF/db/dbNSFP/dbNSFP2.4.txt.gz"
-phastConsDB	SnpEff specific option to describe the full path to the location phastCons directory used by SnpSift<br>Ex: "$SNPEFF/db/phastCons"
-uniprot	Name of the UniProt sequence file (optional, only use if PPH2 is installed)<br>Ex: HUMAN.fasta.gz (default)
-refseq	Name of the RefSeq sequence file (optional, only use if PPH2 is installed)<br>Ex: human.protein.faa.gz (default)
-hgmdUser	HGMD User login (optional, only use if you have an HGMD license)
-hgmdPasswd	HGMD User password (optional, only use if you have an HGMD license)

The following options are provided to adapt the scoring scheme to the users:

-S_Known	Known mutation as annotated by HGMD and/or dbSNP (rsClinicalSignificance="pathogenic/probable-pathogenic").<br>Default: 110
-S_Nonsense	A single-base substitution in DNA resulting in a STOP codon (TGA, TAA or TAG).<br>default: 100
-S_Fs	Exonic insertion/deletion of a non-multiple of 3bp resulting often in a premature stop in the reading frame of the gene.<br>default: 100
-S_EssentialSplice	Mutation in one of the canonical splice sites resulting in a significant effect on splicing (at least 2 out of the 3 programs indicate a relative variation in their score compared to the wild type sequence)<br>default: 90
-S_StartLoss	Mutation leading to the loss of the initiation codon (Met).<br>default: 80

-S_StopLoss Mutation leading to the loss of the STOP codon.
default: 80

-S_CloseSplice Mutation outside of the canonical splice sites (donor site is -3 to +6', acceptor site -12 to +2) resulting in a significant effect on splicing (at least 2 out of the 3 programs indicate a relative variation in their score compared to the wild type sequence).
default: 70

-S_Missense A single-base substitution in DNA not resulting in a change in the amino acid.
default: 50

-S_Inframe Exonic insertion/deletion of a multiple of 3bp.
default: 40

-S_DeepSplice Intronic mutation resulting in a significant effect on splicing (at least 2 out of the 3 programs indicate a relative variation in their score compared to the wild type sequence).
default: 25

-S_Synonymous A single-base substitution in DNA not resulting in a change in the amino acid.
default: 10

-B_phastCons Each variant score is adjusted if a conservation at the genomic level is observed (PhastCons cutoff >0.95)
default: 5

-B_SIFT Missenses scores are adjusted for each SIFT deleterious prediction
default: 5

-B_PPH2 Missenses scores are adjusted for each PPH2 deleterious prediction
default: 5

7. Annotations columns available in the output files

==

In the following table, we describe the annotations that are available in the VaRank output files. It is to notice that , since VaRank can be configured using 2 different annotation engines, in some cases specific annotations are only present while using one annotations engine and in some cases the values for the same type of information are slightly different.

Column name	Annotation	Alamut Batch	SnpEff
**VariantID**	Variant identifier [#chr]_[genomicposition]_[RefBase]_[VarBase]	X	X
**Gene**	Gene symbol	X	X
**omimId**	OMIM® id	X	
**TranscriptID**	RefSeq transcript id	X	
**TranscriptLength**	Length of transcript (full cDNA length)	X	
**Chr**	Chromosome of variant	X	X
**Start**	Start position of variant	X	X
**End**	End position of variant	X	X
**Ref**	Nucleotide sequence in the reference genome (restricted to 50bp)	X	X
**Mut**	Alternate nucleotide sequence (restricted to 50bp)	X	X
**Uniprot**	Uniprot	X	X
**protein**	Protein id (NCBI)	X	
**posAA**	Amino acid position	X	X
**wtAA_1**	Reference codon	X	X
**varAA_1**	Alternate codon	X	X

**Phred_QUAL**	**QUAL:** The Phred scaled probability that a REF/ALT polymorphism exists at this site given sequencing data. Because the Phred scale is -10 * log(1-p), a value of 10 indicates a 1 in 10 chance of error, while a 100 indicates a 1 in 10^10 chance. These values can grow very large when a large amount of NGS data is used for variant calling.	X	X
**HomHet**	Homozygote or heterozygote status	X	X
**TotalReadDepth**	Total number of reads covering the position	X	X
**VarReadDepth**	Number of reads supporting the variant	X	X
**%Reads_variation**	Percent of reads supporting variant over those supporting reference sequence/base	X	X
**VarType**	Variant Type (substitution, deletion, insertion, duplication, delins)	X	X
**CodingEffect**	Variant Coding effect (synonymous, missense, nonsense, in-frame, frameshift, start loss, stop loss)	X	X
**VarLocation**	Variant location (upstream, 5'UTR, exon, intron, 3'UTR, downstream)	X	X
**Exon**	Exon (nearest exon if intronic variant)	X	X
**Intron**	Intron	X	X
**gNomen**	Genomic-level nomenclature	X	
**cNomen**	cDNA-level nomenclature	X	X
**pNomen**	Protein-level nomenclature	X	X
**rsID**	dbSNP variation	X	X
**rsValidation**	dbSNP validated status	X	
**rsClinicalSignificance**	dbSNP variation clinical significance	X	
**rsAncestralAllele**	dbSNP ancestral allele	X	
**rsHeterozygosity**	dbSNP variation average heterozygosity	X	
**rsMAF**	dbSNP variation global Minor Allele	X	
**rsMAFAllele**	dbSNP variation global minor allele	X	
**rsMAFCount**	dbSNP variation sample size	X	
**1000g_AMR_AF**	1000 genomes allele frequency in American population	X	X
**1000g_AFR_AF**	1000 genomes allele frequency in African population	X	X
**1000g_SAS_AF**	1000 genomes allele frequency in South Asian population	X	X
**1000g_EAS_AF**	1000 genomes allele frequency in East Asian population	X	X
**1000g_EUR_AF**	1000 genomes allele frequency in European population	X	X
**1000g_AF**	1000 genomes global allele frequency	X	X
**espRefEACount**	ESP reference allele count in European American population	X	
**espRefAACount**	ESP reference allele count in African American population	X	
**espRefAllCount**	ESP reference allele count in all population	X	
**espAltEACount**	ESP alternate allele count in European American population	X	
**espAltAACount**	ESP alternate allele count in African American population	X	
**espAltAllCount**	ESP alternate allele count in all population	X	
**espEAMAF**	Minor allele frequency in European American population	X	X
**espAAMAF**	Minor allele frequency in African American population	X	X
**espAllMAF**	Minor allele frequency in all population	X	
**espAvgReadDepth**	Average sample read Depth	X	
**delta MESscore (%)**	% difference between the splice score of variant with the score of the reference base	X	
**wtMEScore**	WT seq. MaxEntScan score	X	

**varMEScore**	Variant seq. MaxEntScan score	X	
**delta SSFscore (%)**	% difference between the splice score of variant with the score of the reference base	X	
**wtSSFScore**	WT seq. SpliceSiteFinder score	X	
**varSSFScore**	Variant seq. SpliceSiteFinder score	X	
**delta NNSscore (%)**	% difference between the splice score of variant with the score of the reference base	X	
**wtNNSScore**	WT seq. NNSPLICE score	X	
**varNNSScore**	Variant seq. NNSPLICE score	X	
**DistNearestSS**	Distance to Nearest splice site	X	
**NearestSS**	Nearest splice site	X	
**localSpliceEffect**	Splicing effect in variation vicinity (New donor Site, New Acceptor Site, Cryptic Donor Strongly Activated, Cryptic Donor Weakly Activated, Cryptic Acceptor Strongly Activated, Cryptic Acceptor Weakly Activated)	X	
**SiftPred**	SIFT prediction	X	X
**SiftWeight**	SIFT score ranges from 0 to 1. The amino acid substitution is predicted damaging is the score is <= 0.05, and tolerated if the score is > 0.05.	X	
**SiftMedian**	SIFT median ranges from 0 to 4.32. This is used to measure the diversity of the sequences used for prediction. A warning will occur if this is greater than 3.25 because this indicates that the prediction was based on closely related sequences. The number should be between 2.75 and 3.5	X	
**PPH2pred**	PolyPhen-2 prediction using HumVar model are either "neutral, possibly damaging, probably damaging" or "neutral, deleterious" depending on the annotation engine.	X[1]	X
**phyloP**	phyloP	X	
**PhastCons**	phastCons score	X	X
**GranthamDist**	Grantham distance	X	
**VaRank_VarScore**	Prioritization score according to VaRank	X	X
**AnnotationAnalysis**	Yes or No indicates if the variation could annotated by any annotation engine	X	X
**Avg_TotalDepth**	Total read depth average at the variant position for all samples analyzed that have the variation	X	X
**SD_TotalDepth**	Standard deviation associated with Avg_TotalDepth	X	X
**Count_TotalDepth**	Number of samples considered for the average total read depth	X	X
**Avg_SNVDepth**	Variation read depth average at the variant position for all samples analyzed that have the variation	X	X
**SD_SNVDepth**	Standard deviation associated with Avg_SNVDepth	X	X
**Count_SNVDepth**	Number of samples considered for the average SNV read depth	X	X
**familyBarcode**	Homozygote or heterozygote status for the sample of interest and its associated samples	X	X
**Barcode**	Homozygote or heterozygote status for all sample analyzed together (Hom: 2 ; Het: 1; Sample name is given at the first line of the file: ## Barcode)	X	X
**Hom_Count**	Number of homozygote over all samples analyzed together	X	X
**Het_Count**	Number of heterozygote over all samples analyzed together	X	X
**Allele_Count**	Number of alleles supporting the variant	X	X
**Sample_Count**	Total number of samples	X	X
**SnpEff_Ann**	SnpEff functional annotations information		X
**SnpEff_LOF**	Loss of Function (LOF) assessment (estimated by SnpEff)		X
**SnpEff_NMD**	Nonsense mediate decay (NMD) assessment (estimated by SnpEff)		X

[1] if PPH2 is installed separately.

When –vcfinfo is set to “yes”, all the vcf annotations are reported in separate columns after the last columns described here.

8. FAQ
======

Q: What are the WARNINGs that VaRank mention while running?
A: VaRank writes to the standard output progress of the analysis including warnings about issues or missing information that can be either blocking or simply informative. More specifically while loading the VCF file(s) specific information are under survey such as vcf format consistency, patient redundancy, the total and variant read depth, the genotype, the indels. Any surveyed default will be reported to the user.

Q: I want to run a VaRank analysis again, what shall I do?
A: Simply remove all output files (*.tsv) and type the new command line. All annotations will be kept and the analysis should be done very quickly.

Q: I have already computed 5 samples in my analysis and I want to add 10 more, what should I do?
A: Considering no updated version of any annotation source or VaRank available, you can simply add the new vcf files to the already computed ones, remove all output files (*.tsv), remove simply the /Alamut/AlamutInputFile_all.txt (that will be recreated with the new variants if any) (and PPH2 input file if PPH2 is installed) and rerun VaRank. VaRank will only recompute the missing annotations and will save you the computation time of reannotating multiple times the same variants.

Q: How are the variant homozygous or heterozygous status reported?
A: VaRank trust by default the zygosity status provided by the vcf and report this in the column "*Zigosity*" in the output files. Nonetheless, in the case when no data is provided but total and variant depth of coverage is available, VaRank recompute this by applying the simple rule everything >=Homcutoff (default 80% see options) is homozygous and the rest is heterozygous. In order to clearly show difference with other variants those recomputed will be noted "hom?" or "het?". The same rule is applied when using the option "-Homstatus" except that variant are noted "hom" or "het".

Q: In the output files some values are set to “NA”?
A: When for a specific type of annotation no information is available then the empty value is set to "NA" (e.g. Not Available). Exception is made for several numerical columns (including *rsMAF, espEAMAF, espAAMAF, espAllMAF*) where "-1" is used that allows the user to further filter information without losing data.

Q: What PolyPhen2 prediction are running?
A: Depending on the annotation engine PPH2 either needs to be installed separately (Alamut Batch) or is already integrated (SnpEff). Nevertheless one can still have SnpEff installed and a local installation of PPH2. If the 2 programs are installed and properly setup for the use in VaRank, despite the fact that SnpEff annotations might already contain PPH2 predictions, the local PPH2 installation will be used. If this is not your intention simply unset PPH2 environment variable
