# Supplementary material for: VaRank: a simple and powerful tool for ranking genetic variants
Source: PeerJ. 2015 Mar 3;3:e796. doi: 10.7717/peerj.796 (PMC4358652; doi:10.7717/peerj.796)
Supplement: Supplemental Information 3 [file peerj-03-796-s003.pdf]

# VaRank Tutorial

---

Version 1.0
VaRank is a program for genetic Variant Ranking from NGS data

Copyright (C) 2015 GEOFFROY Véronique, MULLER Jean

Please feel free to contact us for any suggestions or bug reports
email: veronique.geoffroy@inserm.fr; jeanmuller@unistra.fr

================================================================================
TABLE OF CONTENTS
================================================================================
1. INTRODUCTION

2. RUNNING THE EXAMPLE

3. ANALYZING THE EXAMPLE
================================================================================

1. INTRODUCTION
===============

VaRank is a program designed for variant ranking from next generation sequencing data. It provides a comprehensive workflow for annotating and ranking SNVs and indels. In this document we will guide you through the use of VaRank with an example.

In this tutorial, we have used VaRank 1.1.2, Alamut-batch 1.3.1 and the corresponding database (November 2014) and one vcf file. The output files are available on the website (http://www.lbgi.fr/VaRank/).

2. RUNNING THE EXAMPLE
=====================

Assuming that VaRank is properly installed and functional, we will now go through a typical project. The proposed example is extracted from the paper written by Böhm *et al* published in 2013 in the American Journal of Human Genetics. They used Whole Exome Sequencing (WES) to identify a novel gene responsible of tubular aggregates myopathy. The sequencing has been performed on the 4 members of the family (3 affected and one healthy parent) as presented in the following figure:

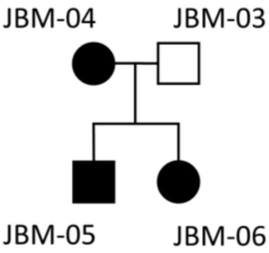


The first step is to download the input file (http://www.lbgi.fr/VaRank/). A single vcf file is available combining the exome data for all 4 patients (JBM-06, JBM-05, JBM-03 and JBM-04).

Then we will create the project directory that will contain the input files (.vcf or .vcf.gz), the working files (annotations files) and the output files (.tsv and .log). After selecting the proper location of your data:

```
mkdir TestExome
cd TestExome
```

Copy the vcf files into the project directory:

```
cp /WHERETHEVCFFILESARESTORED/*.vcf /TestExome
```

VaRank can work with gzipped vcf files, so if the vcf is not compressed one can save disk space already by running the following command line on your vcf files:

```
gzip -9 *.vcf
```

Copy the configuration file example from the VaRank installation directory to the current directory:

```
cp $VARANK/configfile .
```

To be active the configfile must be located in the same directory where the vcf files are stored. The configfile can be either used to change the running parameters of VaRank or to define link between samples.

An example of one default configfile is given here:

```
# This file is used to simplify the configuration of VaRank.
# Anything behind a hashtag is considered as a comment
# Please, feel free to change the VaRank options.

#---------------
# Family Barcode
#---------------
#Grouping sample names together help grouping the naming of the files with the same prefix (fam1_
#for all family members) and to define automatically a specific barcode, the "familyBarcode".
#As an example, 2 families where the fam1 corresponds to a trio sequencing (proband and parents)
#and fam2 with 2 affected child.

#fam1: Sample1 Sample2 Sample3
#fam2: Sample4 Sample5

#----------------
# VaRank Options:
#----------------

#-vcfInfo:              no
#-metrics:              us
#-nowebsearch:          yes
#-rsfromvcf:            no
#-Homstatus:            no
#-Homcutoff:            80
#-SSFcutoff:            -5
#-NNScutoff:            -10
#-MEScutoff:            -15
#-phastConsCutoff:      0.95
#-readFilter:           10
#-readPercentFilter:    15
#-depthFilter:          10
#-freqFilter:           0.01
#-rsFilter:             removeNonPathoRS
#-S_Known:              110
#-S_Fs:                 100
#-S_Nonsense:           100
#-S_EssentialSplice:    90
#-S_StartLoss:          80
#-S_StopLoss:           80
#-S_CloseSplice:        70
#-S_Missense:           50
#-S_DeepSplice:         25
#-S_Inframe:            40
#-S_Synonymous:         10
#-B_phastCons:           5
#-B_SIFT:                5
#-B_PPH2:                5
```

In our example we will add the following line to the configfile to ensure that VaRank will consider the 4 samples together. This will permit the use of the “family barcode” column (a barcode representing only a subset of samples) in addition to the more general Barcode (all samples analyzed together). Moreover the order is important as it will be the exact same in the family barcode.

```
fam1: JBM-06 JBM-05 JBM-04 JBM-03
```

You can now run directly VaRank by using the following command line:

```
VaRank -vcfdir /TestExome >& VaRank_TestExome.log
```

This command will output the normal and error output to a log file. This log file will be used to monitor the VaRank process.

If you are using a queuing system such as slurm you could write the following commands in a file (i.e. cmd.exome.sh):

```
#!/bin/sh
VaRank -vcfdir /TestExome >& VaRank_TestExome.log
```

and run it on the cluster:

```
sbatch cmd.exome.sh
```

The log file (here VaRank_TestExome.log) contains very useful information that should be checked systematically to assess the proper run of VaRank. This includes among other, the used Tcl/Tk version (Line 1), VaRank's version (L2), the parameters used to setup the current VaRank run (L18-L57). It gives also a report for each major running steps of VaRank: parsing of the vcf file (L59-L63) and extraction of the variants, annotation (here via Alamut Batch L64-L70), scoring of the variants (L72-L73), writing of the output files (L74-L85) and a final ending statement (L86).
It will also contain any warnings or error messages in the case of an unusual situation or crash. As an example in this log file, VaRank has been used with a not yet supported version of Alamut (L67-L68).

```
Tcl/Tk version: 8.6
VaRank 1.2
VaRank is a program for Ranking genetic Variation from NGS data

Copyright (C) 2015 GEOFFROY Veronique and MULLER Jean

Please feel free to contact us for any suggestions or bug reports
email: veronique.geoffroy@inserm.fr; jeanmuller@unistra.fr

...downloading the configuration data (January 13 2015 - 22:30)
	...configuration data by default
	...configuration data from /TestExome/configfile
	...configuration data given in arguments
	...checking configuration data
	******************************************
	VaRank has been run with these arguments :
	******************************************
	-B_PPH2 5
	-B_SIFT 5
	-B_phastCons 5
	-DB /VaRank/Databases
	-Homcutoff 80
	-Homstatus no
	-MEScutoff -15
	-NNScutoff -10
	-SSFcutoff -5
	-S_CloseSplice 70
	-S_DeepSplice 25
	-S_EssentialSplice 90
	-S_Fs 100
	-S_Inframe 40
	-S_Known 110
	-S_Missense 50
	-S_Nonsense 100
	-S_StartLoss 80
	-S_StopLoss 80
	-S_Synonymous 10
	-Version 1.2
	-alamutDir /Software/Alamut/alamut
	-depthFilter 10
	-extann
	-freqFilter 0.01
	-hgmdPasswd
	-hgmdUser
	-metrics fr
	-nowebsearch yes
	-phastConsCutoff 0.95
	-pph2Dir
	-readFilter 10
	-readPercentFilter 15
	-refseq human.protein.faa.gz
	-rsFilter removeNonPathoRS
	-rsFromVCF no
	-sourcesDir / VaRank/sources
	-uniprot HUMAN.fasta.gz
	-vcfDir /TestExome
	-vcfInfo no
	******************************************
...parsing the VCF file (/TestExome/Exomes_STIM1.vcf.gz) (January 13 2015 - 22:30)
	File loaded: 76031 variation(s) and 4 sample(s) (January 13 2015 - 22:30).
	File loaded: Total Read Depth:  304124 variant(s) are different to 0, 0 variant(s) are equal to 0, 0 are empty
	File loaded: Allele Read Depth: 304122 variant(s) are different to 0, 2 variant(s) are equal to 0, 0 are empty
...VCF file(s) loaded: 1 file(s) for 76031 variation(s) in 4 sample(s) (January 13 2015 - 22:30)
...creation of the alamut input file (/TestExome/Alamut/AlamutInputFile_all.txt) (January 13 2015 - 22:30)
...running Alamut-Batch (January 13 2015 - 22:30)
################################################################################
```

```
        ...WARNING: VaRank supports version of Alamut-batch until 1.3
          You are using Alamut-batch 1.3.1 (not tested)
################################################################################
...parsing Alamut-Batch results (January 13 2015 - 22:30)
...PPH environment variable not specified, not running PPH2
...scoring each genetic variant (January 13 2015 - 22:31)
...classifying each genetic variant (January 13 2015 - 22:31)
...writing output files: all variants, ranking by var (January 13 2015 - 22:31)
        ...organizing ranking output from alamut data (70785 scores) (January 13 2015 - 22:31)
        ...organizing ranking output from data not analysed by alamut (January 13 2015 - 22:31)
        ...updating VariantID nomenclature for large indels (January 13 2015 - 22:31)
        ...writing "*_allVariants.rankingByVar" output files (January 13 2015 - 22:31)
...writing output files: all variants, ranking by gene (January 13 2015 - 22:31)
        ...searching for all variants (January 13 2015 - 22:31)
        ...scoring of each gene (January 13 2015 - 22:31)
        ...writing "*_allVariants.rankingByGene.tsv" output files (January 13 2015 - 22:32)
...writing all filtered ranking files (January 13 2015 - 22:32)
...writing patients and global statistics (January 13 2015 - 22:32)
...VaRank Statistics: 76031 variation(s): 12735 homozygous, 45084 heterozygous, 18212 both and 0 empty
...VaRank is done with the analysis (January 13 2015 - 22:32)
```

Once VaRank has finished running, you need to make sure that the log file does not contain errors and check the warnings if any. The output directory (/TestExome/) should contain several files according to the organization described below:

```
/TestExome/
 |----- configfile                                   |
 |----- *InputFile.vcf.gz                            #Input files
 |
 |----- Alamut/                                      #Contains all Alamut Batch related files
 |      |----- AlamutInputFile_all.txt               #Alamut input file generated from the vcf(s) files
 |      |----- AlamutAnnotations_all.txt             #Alamut output file with annotated variants
 |      |----- AlamutUnnanotated_all.txt             #Alamut output file with unannotated variants
 |      |----- AlamutOutput_all.txt                  #Alamut log file
 |
 |----- PPH2/                                        #(optional) Contains all PolyPhen-2 related files
 |      |----- PPH2input_all.txt                     #PPH2 input file
 |      |----- PPH2features_all.txt                  #PPH2 output file
 |      |----- PPH2humvar_all.txt                    #PPH2 output file
 |      |----- PPH2errors_all.txt                    #PPH2 log file
 |
 |----- fam#_SampleName_allVariants.rankingByVar.tsv
 |----- fam#_SampleName_filteredVariants.rankingByVar.tsv
 |
 |----- fam#_SampleName_allVariants.rankingByGene.tsv
 |----- fam#_SampleName_filteredVariants.rankingByGene.tsv
 |
 |----- fam#_SampleName_statistics.tsv               #Short counts report (e.g. homozygous, heterozygous
 |                                                   #and total counts) for each of the variant categories
 |
 |----- SNV_global_statistics.tsv                    #Contains the same counts as defined for each patient
 |                                                   #but for the whole analyzed cohort
```

So after the run you should end up with the following output files:

```
fam1_JBM-03_allVariants.rankingByGene.tsv
fam1_JBM-03_allVariants.rankingByVar.tsv
fam1_JBM-03_filteredVariants.rankingByGene.tsv
fam1_JBM-03_filteredVariants.rankingByVar.tsv
fam1_JBM-03_statistics.tsv
fam1_JBM-04_allVariants.rankingByGene.tsv
fam1_JBM-04_allVariants.rankingByVar.tsv
fam1_JBM-04_filteredVariants.rankingByGene.tsv
fam1_JBM-04_filteredVariants.rankingByVar.tsv
fam1_JBM-04_statistics.tsv
fam1_JBM-05_allVariants.rankingByGene.tsv
```

fam1_JBM-05_allVariants.rankingByVar.tsv
fam1_JBM-05_filteredVariants.rankingByGene.tsv
fam1_JBM-05_filteredVariants.rankingByVar.tsv
fam1_JBM-05_statistics.tsv
fam1_JBM-06_allVariants.rankingByGene.tsv
fam1_JBM-06_allVariants.rankingByVar.tsv
fam1_JBM-06_filteredVariants.rankingByGene.tsv
fam1_JBM-06_filteredVariants.rankingByVar.tsv
fam1_JBM-06_statistics.tsv
SNV_global_statistics.tsv
VaRank_TestExome.log

5 output files for each sample submitted (ranking either by gene or by variants each filtered or not and a statistics file), 1 global statistics file (SNV_global_statistics.tsv) and the log file (VaRank_TestExome.log).

## 3. ANALYZING THE EXAMPLE

========================

A first look at the global statistics of the project is helpful to check if the data generated by the experiment are in a good range. The SNV_global_statistics.tsv file classify the non-redundant count of each samples variations using functional categories. As an example the sequencing of the 4 exomes generated 76031 non redundant variants and each sample has on average 48098 variants. These numbers seems pretty standard for a WES.

| What | Total | Mean | SD |
|---|---|---|---|
| synonymous | 15481 | 10797 | 404 |
| missense | 13813 | 8789 | 207 |
| nonsense | 205 | 84 | 22 |
| In-frame | 444 | 226 | 13 |
| Frameshift | 1000 | 363 | 28 |
| startloss | 25 | 15 | 2 |
| stoploss | 17 | 12 | 1 |
| unknown | 5246 | 3146 | 280 |
| intron | 33605 | 20980 | 1322 |
| upstream | 1001 | 585 | 64 |
| 5'UTR | 1040 | 581 | 67 |
| 3'UTR | 1788 | 1105 | 78 |
| downstream | 1098 | 654 | 56 |
| splice site | 0 | 0 | 0 |
| Total | 76031 | 48098 | 2491 |

We will now have a quick look at the one of the samples output files. The .tsv files are tab separated values formatted files that can be open in any spreadsheet program such as “Microsoft Excel” or “OpenOffice Calc”. Each line represent another variant and each column a specific annotation. Each sample should have the following list of files:
fam1_JBM-05_allVariants.rankingByGene.tsv
fam1_JBM-05_allVariants.rankingByVar.tsv
fam1_JBM-05_filteredVariants.rankingByGene.tsv
fam1_JBM-05_filteredVariants.rankingByVar.tsv
fam1_JBM-05_statistics.tsv

The “allVariants” and “filteredVariants” contain the same annotation columns but the second file has less variants. Some variants have been filtered out according to several criteria in to simplify the analysis. Most of the filters can be redone using the “allVariants” file. In this example, JBM-05 has 2254 variants stored in the “filteredVariants” file and 44336 variants in the “allVariants” file.

The “filteredVariants” files are already prefiltered for variation frequency (default is to keep <1%), sequence quality (keep if variant depth and total depth of coverage >10, percent of reads supporting variant >15%). The variant with a validated annotation in the dbSNP database (i.e. at least with 2 evidence supporting the variation including multiple independent submissions, frequency or genotype data, submitter confirmation, observation of all alleles in at least two chromosomes, genotyped by HapMap, and present in the 1000G project) but that are not pathogenic (from the ClinicalSignificance field in dbSNP) are removed.

We will not go through all of the annotation columns which are already described in the reference manual of VaRank (http://www.lbgi.fr/VaRank/) but focus on the use of the barcode.

Starting from the following file:
`fam1_JBM-05_filteredVariants.rankingByVar.tsv`

The first 2 lines of each file describe the list of samples used to compute the barcode and the family barcode:

```
## Barcode: JBM-06 JBM-05 JBM-04 JBM-03
## FamilyBarcode: JBM-06 JBM-05 JBM-04 JBM-03
```

The barcode and family barcode are the same in this example but in reality you could run multiple exomes at once and the barcode will represent the total list of samples analyzed and the family barcode only the one that you have decided to group together.

Following this information, we find the header line with a simple column name describing each annotation and then a single line for each variant.

| VariantID | Gene | omimId | TranscriptID | TranscriptLength | Chr | … |
|---|---|---|---|---|---|---|
| 12_122295335_T_C | HPD | 609695 | NM_001171993.1 | 1883 | 12 | … |

The following table illustrate the basic information that can be used to filter the results. You can see here the 10 first variants ranked by VaRank.

| VariantID | Gene | TranscriptID | Chr | Start | Ref | Mut | Zygosity | TotalRead Depth | VarRead Depth | cNomen | pNomen | rsID | rsValidation | rsClinicalSignificance | rsMAF | espAllMAF | SiftPred | VaRank_ VarScore | familyBarcode | Barcode | #Hom | #Het | #Allele | #Sample |
|---|---|---|---|---|---|---|---|---|---|---|---|---|---|---|---|---|---|---|---|---|---|---|---|---|
| 16_56548501_C_T | BBS2 | NM_031885.3 | 16 | 56548501 | C | T | hom | 13 | 13 | c.209G>A | p.= | rs4784677 | Cluster/Frequency /HapMap/1000G | pathogenic | 0,004 | 0,00554017 | NA | 110 | '2222' | '2222' | 4 | 0 | 8 | 4 |
| 5_131729380_G_A | SLC22A5 | NM_003060.3 | 5 | 131729380 | G | A | het | 32 | 13 | c.1463G>A | p.Arg488His | rs28383481 | Cluster/Frequency /HapMap/1000G | notprovided,pathogenic | 0,004 | 0,00407504 | Deleterious | 110 | '1101' | '1101' | 0 | 3 | 3 | 4 |
| 22_29121087_A_G | CHEK2 | NM_001005735.1 | 22 | 29121087 | A | G | het | 31 | 16 | c.599T>C | p.Ile200Thr | rs17879961 | Cluster/Frequency /1000G | likelypathogenic,pathog enic,riskfactor | 0,002 | 0,00161489 | Deleterious | 110 | '1101' | '1101' | 0 | 3 | 3 | 4 |
| 12_103234285_G_A | PAH | NM_000277.1 | 12 | 103234285 | G | A | het | 45 | 18 | c.1208C>T | p.Ala403Val | rs5030857 | Cluster/Frequency /1000G | pathogenic | 0,001 | 0,000384438 | Deleterious | 110 | '0101' | '0101' | 0 | 2 | 2 | 4 |
| 11_3988893_A_G | STIM1 | NM_001277961.1 | 11 | 3988893 | A | G | het | 28 | 11 | c.251A>G | p.Asp84Gly | rs397514675 | NA | pathogenic | 0 | -1 | Deleterious | 110 | '1110' | '1110' | 0 | 3 | 3 | 4 |
| 7_117230454_G_C | CFTR | NM_000492.3 | 7 | 117230454 | G | C | het | 23 | 11 | c.1727G>C | p.Gly576Ala | rs1800098 | Cluster/Frequency /Submitter/1000G | uncertainsignificance,pa thogenic | 0,004 | 0,00523399 | Tolerated | 110 | '0110' | '0110' | 0 | 2 | 2 | 4 |
| 8_52733231_G_A | PCMTD1 | NM_052937.2 | 8 | 52733231 | G | A | het | 35 | 13 | c.754C>T | p.Arg252* | rs75748152 | Cluster | notprovided | 0 | -1 | NA | 105 | '0110' | '0110' | 0 | 2 | 2 | 4 |
| 17_16068343_G_A | NCOR1 | NM_006311.3 | 17 | 16068343 | G | A | het | 34 | 13 | c.568C>T | p.Arg190* | rs78230791 | Cluster | NA | 0 | -1 | NA | 105 | '0101' | '0101' | 0 | 2 | 2 | 4 |
| 2_112614429_G_A | ANAPC1 | NM_022662.3 | 2 | 112614429 | G | A | het | 34 | 20 | c.1393C>T | p.Gln465* | rs72936240 | Cluster | NA | 0 | -1 | NA | 105 | '1111' | '1111' | 0 | 4 | 4 | 4 |
| 21_10942756_G_A | TPTE | NM_199261.3 | 21 | 10942756 | G | A | het | 123 | 45 | c.685C>T | p.Arg229* | NA | NA | NA | -1 | -1 | NA | 105 | '1101' | '1101' | 0 | 3 | 3 | 4 |

In order to keep data visible on a single page, the last columns (Hom_Count, Het_Count, Allele_Count, Sample_Count) have been renamed.

To further describe the barcode, one can look at the first variant in *BBS2* and easily understand that the Barcode “2222” indicates that this variant is present in all 4 members sequenced at the homozygous state.

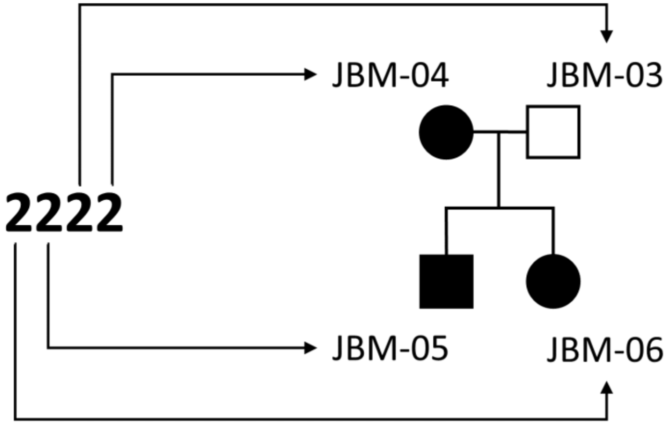

In the case of our example, the family has 3 affected patients (mother and 2 children) and one healthy parent. This configuration strongly suggest a dominant mutation that should be absent from the father. Testing this hypothesis is fairly easy thanks to the use of the family barcode. Reminding the order of the barcode (3 affected first and the healthy parent at the end) one should look for barcode like this: “1110”, which means that the 3 affected should be heterozygous for the variation and that the variant should be absent in the father.
Applying this further reduces the number of filtered variants from 2254 to 125. The top first remaining variant is a mutation in *STIM1* (the mutation is also present in the previous table, 5$^{th}$ position) labeled pathogenic in dbSNP and associated by the author of the paper with the disease in this family.
